# The Secret Lives of Fluorescent Membrane Probes as Revealed by Molecular Dynamics Simulations

**DOI:** 10.3390/molecules25153424

**Published:** 2020-07-28

**Authors:** Hugo A. L. Filipe, Maria João Moreno, Luís M. S. Loura

**Affiliations:** 1 Chemistry Department, Coimbra Chemistry Center, Faculty of Sciences and Technology, University of Coimbra, 3004-535 Coimbra, Portugal; hlfilipe@uc.pt; 2 Coimbra Chemistry Center and CNC—Center for Neuroscience and Cell Biology, Chemistry Department, Faculty of Sciences and Technology, University of Coimbra, 3004-535 Coimbra, Portugal; mmoreno@ci.uc.pt; 3 Coimbra Chemistry Center and CNC—Center for Neuroscience and Cell Biology, Faculty of Pharmacy, University of Coimbra, 3000-548 Coimbra, Portugal

**Keywords:** fluorescence, lipid bilayer, membrane probes, molecular dynamics, molecular simulation

## Abstract

Fluorescent probes have been employed for more than half a century to study the structure and dynamics of model and biological membranes, using spectroscopic and/or microscopic experimental approaches. While their utilization has led to tremendous progress in our knowledge of membrane biophysics and physiology, in some respects the behavior of bilayer-inserted membrane probes has long remained inscrutable. The location, orientation and interaction of fluorophores with lipid and/or water molecules are often not well known, and they are crucial for understanding what the probe is actually reporting. Moreover, because the probe is an extraneous inclusion, it may perturb the properties of the host membrane system, altering the very properties it is supposed to measure. For these reasons, the need for independent methodologies to assess the behavior of bilayer-inserted fluorescence probes has been recognized for a long time. Because of recent improvements in computational tools, molecular dynamics (MD) simulations have become a popular means of obtaining this important information. The present review addresses MD studies of all major classes of fluorescent membrane probes, focusing in the period between 2011 and 2020, during which such work has undergone a dramatic surge in both the number of studies and the variety of probes and properties accessed.

## 1. Introduction

Fluorescent molecules have been very important tools in the characterization of biomembrane properties since the late 1960s [1,2,3,4,5]. They have been widely used to study structural properties such as electrostatics, polarity, and phase behavior, as well as membrane dynamics such as fluidity, and membrane fusion/budding. The sensitivity of fluorescence to the polarity and fluidity of the medium where they are embedded allows a detailed study of membrane micro-heterogeneity [6], which is of utmost importance in its role in cell structure and function. Fluorescence Resonance Energy Transfer (FRET), being sensitive to the relative distance and orientation of the donor and the acceptor molecules, is an additional fluorescence methodology of broad application in the study of biomembranes [7]. To obtain quantitative information from those methodologies, it is necessary to know the location and orientation of the fluorescent probes when embedded in the membrane, as well as the local perturbation of the membrane due to the presence of the fluorescent molecules. This knowledge about bilayer-inserted probe behavior should be obtained using independent techniques, as suggested very early on [8]. However, those molecular details are very difficult to obtain from experimental bulk measurements, and, more recently, atomic level approaches such as Molecular Dynamics (MD) simulations have gained fundamental importance.

Fluorescence molecules also play a very important role in the understanding and ultimately in the prediction of small molecule bioavailability. The major function of biomembranes is to delimit the aqueous media of tissues, cells, and cell organelles. To reach its target location, a nutrient, drug or biological ligand must interact with and permeate through several biomembranes. Fluorescence- based approaches are very promising in this respect, due to the very high sensitivity and versatility of the methodologies available. It is therefore unsurprising that most studies on the characterization of the kinetics of small molecule/membrane interactions have involved fluorescent molecules, them being the molecules of interest, or tools to follow the relevant molecule. Here again, the details provided by MD simulations are of very high importance to understand the relation between the observed bioavailability and the structure of the permeating molecule. The most important properties in this respect are the location and orientation in the membrane, the interactions established, and the local perturbation of the membrane [9,10,11]. Nowadays, computational chemistry tools, boosted by a larger computational power, evolved to a level that makes these techniques completely suited to the characterization of many properties of biomolecular systems, such as their preferred organization and free energies of molecular processes. MD simulations are a valuable tool for this purpose, as all details of the interactions may be established [9,10], and have been used to obtain both detailed atomic-scale and coarse-grained (CG) level information on the behavior of fluorescent probes with lipid bilayers.

This paper examines the applications of MD to the study of fluorescent membrane probes, picking up from our last reviews on this subject [12,13]. Works described in these articles are generally mentioned very briefly here, as we focus on the nine-year period starting in 2011. During this period, a review article about molecular modeling of lipid probes was published [14]. It should be noted that overlap between that review and the present paper is quite small, as the former leaves out a large number of important studies (for example, it excludes sterol probes altogether), while addressing other types of membrane probes (for nuclear magnetic and electron spin resonance spectroscopies).

Historically, the first probes to be simulated in membranes using MD had simple apolar fluorophores, such as 1,6-diphenylhexatriene (DPH) and pyrene. This enabled straightforward parameterization using standard force field atom types. Partial charges on fluorophore atoms only deviate slightly from zero values, and sometimes consideration of null atomic charges in the fluorophore affected the probe behavior only minimally [15,16], rendering their accurate computation less crucial for this class of molecules. As described in our previous papers, starting in the late 2000s, the study of these compounds was followed by simulations of polar fluorophores such as NBD, BODIPY, carbocyanine or rhodamine. This accompanied the emergence of a variety of tools that were developed to aid parameterization for different force fields, such as Automated Topology Builder for GROMOS96 [17,18], CHARMM-GUI for CHARMM [19,20], Antechamber for AMBER [21], and LigParGen for OPLS-AA [22]. In addition to creating compatible topology parameters, these resources also allowed calculation of atom charges using methodologies consistent with the parent force field. If required, atom charges could still be obtained from quantum calculations carried out outside these tools, following the recipes provided in the original force field development papers.

The availability of these parameterization tools, combined with the interest in understanding both the behavior of probes and the effects induced on host membrane properties, has predictably led to a marked increase in the number of computational studies of fluorescence membrane probes, as well as a diversification of the chemical structures of the studied compounds. Notwithstanding, considerable interest still exists on other aspects of classical probes such as DPH or pyrene. A more recent trend, absent in the works that were described in our previous reviews, is the calculation of optical properties of the fluorophores using combined quantum chemical and molecular dynamics studies. Additionally, there has been a surge in studies where biased simulations with enhanced sampling are used to obtain equilibrium and kinetic properties related to the interaction of small molecules (namely drugs) with the bilayers. Those studies provide directly the energy profile for the solute in the aqueous media and at different depths in the membrane, from which the rate of translocation and overall permeation may in principle be calculated [23,24,25].

The text below is divided into sections, each one related to a different class of probes. Within each section or subsection, the first paragraph briefly contextualizes the probe/group of probes addressed and summarizes previous findings, whereas other paragraphs describe related research published since 2011. In this review, we mostly refrain from commenting on force field or simulation parameter choices. We chose to emphasize the power of MD simulation in the characterization (and, increasingly, in the prediction) of the behavior of fluorescent membrane probes, summarizing the main findings in each class of probe compounds. We mostly avoid exhaustive mention of those technical simulation details, to make this paper an accessible and useful read for both computational researchers and experimentalists. More detailed descriptions may be found in the original references. However, it must be emphasized that correct parametrization, simulation and analysis protocols are absolutely crucial for the obtainment of meaningful information, and therefore we duly comment below some instances where we found them visibly questionable.

## 2. Aromatic Hydrocarbon Fluorophores

### 2.1. DPH and TMA-DPH

**Figure 1:**
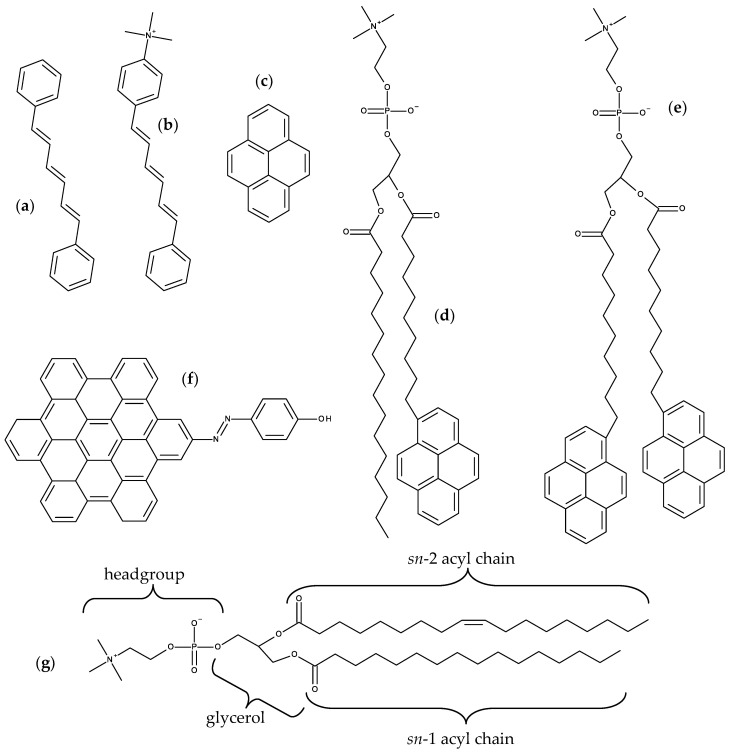
(**a**–**f**) Structures of aromatic hydrocarbon probes. (**a**) DPH; (**b**) TMA-DPH; (**c**) pyrene; (**d**) PYR-PC; (**e**) PYR10; (**f**) HBC-azo. (**g**) Structure of a membrane lipid commonly used in MD simulations (POPC), with indication of different parts of the molecule.

DPH (Figure 1a) is a rod-shaped molecule whose fluorescence polarization is very sensitive to the microenvironment viscosity [26]. Its orientation in the bilayer remained an unresolved question for a long time. From analysis of time-resolved fluorescence anisotropy, a bimodal angular distribution of the long molecular axis was proposed, with one population oriented parallel to the membrane normal, and the other one with a perpendicular alignment, interpreted as corresponding to the presence of DPH in the bilayer midplane, between the two leaflets [27]. The latter scenario was challenged by MD simulation results [28]. When inserted in fluid 1,2-dipalmitoyl-*sn*-3-glycero-phosphocholine (DPPC) [15,29] and DPPC/cholesterol (Chol) [30] bilayers, DPH resides in the acyl chain region of the DPPC bilayer, with a wide distribution of long axis tilt angles θ relative to the bilayer normal. Maximal probability was observed for θ ≈ 25°, indicating rough alignment with the lipid acyl chains. Configurations with long axis parallel to the membrane plane (θ ≈ 90°) were observed, but without correlation with center of mass location near the bilayer center. For bilayers containing 20 mol% Chol, the most significant alteration is that the angular distribution of the DPH long axis becomes narrower, with a single maximum for θ ≈ 10° and essentially zero values for θ ≈ 90°.

Hurjui et al. [31] found that, in unsaturated 1,2-dioleoyl-*sn*-glycero-3-phosphocholine (DOPC) bilayers, a representative population of DPH molecules was intriguingly found in the acyl-chain region of the bilayer immediately beneath the DOPC/water interface, with a tilt angle of near 90° relative to the bilayer normal. No such orientations were found for molecules close to the bilayer center, however.

**Figure 2:**
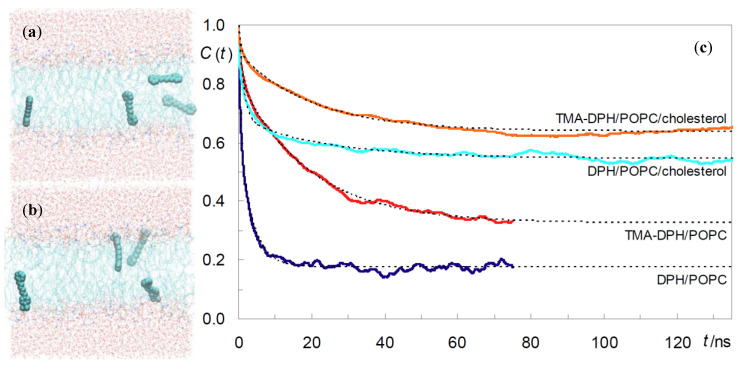
(**a**) Snapshot of a simulation of 4 DPH molecules in a POPC bilayer; (**b**) Same as (**a**), but with 4 TMA-DPH molecules; (**c**) Rotational autocorrelation curves *C*(*t*) for the long axis of DPH and TMA-DPH in POPC and POPC/20 mol% Chol. Dotted lines are biexponential fits with a residual component. Reprinted from [32] with permission from Elsevier. Copyright 2016 Elsevier.

Do Canto et al. [32] simulated both DPH and its trimethylammonium derivative, TMA-DPH (Figure 1b), in 1-palmitoyl-2-oleoyl-*sn*-glycero-3-phosphocholine (POPC) bilayers (Figure 1g), in the absence or in the presence of 20 mol% Chol. For DPH, their results corroborated previous MD efforts, regarding location, orientation, and translocation to the opposite bilayer leaflet. The latter process mostly occurs by vertical translation of the molecule, with the previously most internally located phenyl ring becoming the most externally located one in the opposite leaflet. However, in roughly a quarter of these events DPH translocation is accompanied by rotation around the long axis of the molecule (flip-flop), which at one point is oriented parallel to the bilayer plane (Figure 2a). This probe configuration resembles the scenario of one population oriented perpendicular to the bilayer normal, between the two leaflets. However, these configurations always represented transient states, with DPH rapidly (most often within ~100–200 ps) realigning with the bilayer normal. Additionally, these full rotations are more common far from the bilayer center and are not necessarily accompanied by translocation. They also addressed probe rotation by calculating rotational autocorrelation functions, which were compared to literature time-resolved fluorescence anisotropy data.

Paloncýová et al. [33] expanded on the analysis of the rotational behavior of DPH, employing a previously available model [34] for time-resolved anisotropy, together with a maximum entropy approach, to obtain order parameters <*P*_2_> and <*P*_4_>. Besides POPC, the studied systems included gel-phase DPPC and liquid ordered 2:1 *N*-stearoyl sphingomyelin (SSM)/Chol lipid systems. As expected, rotation is negligible in the gel phase and very limited in SSM/Chol. Interestingly, for the latter system, a relatively stable configuration (residence lifetime ≈10 ns) was observed in the center of the bilayer, with orientation parallel to the membrane plane.

Because DPH is frequently used experimentally to infer about drug effects on membrane fluidity, it is important to gauge whether DPH orientation in these cases reflects only changes in membrane properties or might be affected by the drug molecules present in the bilayer. To this effect, a recent study [35] addressed 10 mol% of itraconazole (ITZ) in a large (1024 lipid molecules) POPC bilayer, also containing 1.5 mol% of DPH. In presence of ITZ, DPH fluorescence anisotropy is reduced (twofold for 20 mol% ITZ) while the fluorescence lifetime increases only slightly (~5% for 20 mol% ITZ), pointing to increased membrane fluidity. However, this is not corroborated by experimental fluorescence recovery after photobleaching experiments, nor from evaluation of lipid properties in MD simulations. Because ITZ molecules lie mostly parallel to the membrane plane, near the interface, they push DPH to a deeper region of the bilayer. Therefore, DPH is not reporting changes in bilayer properties, but rather its own displacement to a more disordered region. This study shows that experimental studies using common membrane probes may be interpreted incorrectly, and that MD simulations may help understanding what is actually happening in such cases.

DPH is employed as a reporter of the hydrophobic core of the bilayer. In contrast, because of its cationic group which purposely acts as an anchor to the water/bilayer interface, TMA-DPH is mainly used to probe the more ordered shallow regions of the bilayer (glycerol backbone and upper segments of the phospholipid acyl chains). This could be expected because the trimethylammonium group of TMA-DPH is identical to the terminal (and most external) charged N(CH_3_)_3_ onium group of phosphatidylcholine. A preliminary MD study was reported for TMA-DPH in fluid DPPC [36], only covering 2 ns of simulation (of which 1 ns was taken as system equilibration). The simulations focused in the atomic positions of the end N and H atoms and revealed undistinguishable positions of the H atoms closest to the bilayer center or interface of DPH and TMA-DPH, when taking into account the statistical uncertainty. However, the exceedingly small simulation time, together with the scarcity of parameterization details presented in the published work, limit considerably the degree of certainty associated with this report. A more definite study was carried out by do Canto et al. [32], who determined that the fluorophore of TMA-DPH is only slightly (by ~0.3–0.4 nm) more external in membranes than that of DPH (Figure 2b). This transverse position allows the cationic TMA moiety to lie between the electronegative lipid phosphate and carbonyl/ester groups, maximizing favorable interactions with both of them. In agreement with experimental findings, TMA-DPH shows hindered rotational and lateral diffusion compared to DPH (see Figure 2c for comparison of rotational autocorrelation functions). However, this difference cannot be justified by the small difference in fluorophore position in the bilayer and arises mostly from the establishment of the above mentioned probe-lipid interactions in TMA-DPH, which are absent in DPH.

### 2.2. Pyrene and Its Derivatives

Pyrene (Figure 1c) is a polycyclic aromatic hydrocarbon with notable spectroscopic properties, including a long fluorescence lifetime (>100 ns in a variety of systems) and ability to form excimers. These distinctive features have led to the use of both free pyrene and pyrene-labeled lipids in membrane biophysics [37]. Previous MD studies of bilayer-inserted free pyrene [38,39] or acyl-chain labeled 1-palmitoyl-2-(1-pyrenedecanoyl)-*sn*-glycero-3-phosphocholine (PYR-PC; Figure 1d) [16] revealed that pyrene has broad transverse location (with maxima at z ~ 1.0 nm for free pyrene and z ~ 0.75 nm for the pyrene lipid probe) and orientation distributions, with minor effects on bulk host bilayer properties (but significant local effects, with ordering of fluid phase and disordering of gel phase).

Loura et al. [40] simulated free pyrene in POPC bilayers containing varying proportions of Chol (up to 40 mol%). Their results confirmed previous findings regarding the Chol-free liquid disordered bilayers. For systems with 20 and 40 mol% Chol, it was found that pyrene induced local disordering (reducing the lipid acyl chain order parameter, especially near the end of the chains) opposing the effect of Chol. Upon increasing Chol concentration in the bilayer, the relative position of pyrene and the POPC carbonyl and phosphocholine groups is invariant, while the local water density around the probe decreases, supporting use of pyrene Ham effect to measure equivalent polarity in lipid bilayers [41]. This was also verified in a subsequent study using *N*-palmitoylsphingomyelin (PSM)/Chol mixtures [42]. Again, the position of pyrene relative to the lipid glycerol/carbonyl moiety is constant for varying Chol content, whereas the local hydration (very low already in the absence of Chol) drops threefold for 20 mol% Chol, and subsequently stays constant up to 50 mol%.

Turning to pyrene-labeled probes, Fraňová et al. [43] addressed the question of where to attach the fluorophore in dipyrenyl-phosphatidylcholine lipids, to minimize the extent of membrane perturbation and to optimize pyrene dimer formation. As well as the parent compound 1,2-di-(1-pyrenedecanoyl)-*sn*-glycero-3-phosphocholine (PYR10; Figure 1e), phosphatidylcholine analogues with shorter acyl chains PYR6 and PYR8 were also considered. Effects of the different probes on host lipid properties are similar and consistent with previous studies (significant locally, modest overall). Pyrene dimers were observed involving fluorophores within the same probe molecule (A), belonging to different molecules in the same bilayer leaflet (B), or in opposite leaflets (C). However, dimers of type B were rare for PYR10 compared to PYR6 and PYR8, whereas dimers of type C were common for PYR10 (in agreement with experimental observations [44]) but entirely absent in the shorter-chained analogues. These dipyrenyl-phosphatidylcholine probes (together with PYR4) have been used in the past to try to measure lateral pressure profiles [45]. In that experimental work, information on this elusive property was inferred from pyrene excimer/monomer fluorescence emission ratios. However, calculations of both lateral pressure and excimer formation rates for bilayers containing PYR4, PYR6, PYR8 and PYR10 showed no correlation between the two properties [46], thus discouraging the usage of dipyrenyl-phosphatidylcholine to measure the shape of the lateral pressure profile.

### 2.3. Hexabenzocoronene Photoswitchable Probes

Azobenzene compounds are notable for their high photoswitching efficiency, based on the reversible *trans* to *cis* isomerization of the N=N bond upon photoexcitation, and the stability of the *cis* metastable state. A recent study addressed the use of a novel azobenzene probe (HBC-azo; Figure 1f), with a hydroxyl group added in the *para* position of one phenyl ring to enhance the polar interaction, and the second phenyl ring replaced by the hexabenzocoronene moiety, to ensure strong interaction with both the polar and the hydrophobic regions of the lipid membrane [47]. Although this is not strictly a hydrocarbon probe, the large aromatic group dominates its structure, hence its inclusion in this section. HBC-azo was simulated in both fluid DOPC and gel DPPC bilayers. Subsequently, a number of uncorrelated snapshots were extracted from the MD simulations, and (after imposing a suitable cutoff) used as input structures for the Quantum Mechanics/Molecular Mechanics (QM/MM) calculations of optical probe properties, including one- and two-photon absorption, and time-resolved fluorescence emission and anisotropy parameters. From MD simulations, the authors observed that although the *trans* isomer has similar position and orientation in both lipid phases, the *cis* conformer shows marked differences. This leads to similar one- and two-photon absorption spectra for the *trans* isomer, but to marked differences in the two phases for the *cis* form. The study illustrates that it is possible to screen between different lipid phases for the same isomer, and that the different isomers in the same phase lead to different responses, suggesting that HBC-azo may be a versatile probe for phase detection.

## 3. Charge-Transfer-Based and Other Solvatochromic Probes

Many fluorescent probes change their emission characteristics in response to fundamental properties of molecular environment, such as polarity, viscosity, and molecular order. The classical environment-sensitive probes are solvatochromic dyes presenting donor and acceptor groups, the so-called push–pull fluorophores, or excited-state intramolecular proton transfer [6]. The popular membrane probes described in this section are included in this group.

### 3.1. NBD Probes

*N*-(7-Nitrobenz-2-oxa-1,3-diazol-4-yl)-(NBD-) labeled derivatives are commercially available for all major phospholipid classes, and have been widely used as fluorescent analogues of lipids in biological and model membranes [48,49]. NBD was the first polar fluorophore to be simulated in lipid bilayers, confirming its snorkeling to the glycerol region when attached to the lipid acyl chain [50,51]. Subsequently, location and orientation data from MD simulations was used for direct estimation of the NBD-NBD Förster Resonance Energy Transfer (FRET) orientation factor, κ^2^ [52]. This latter study served as the basis for the development of a general numerical method for calculation of average κ^2^ values employing information on location and orientation obtained from MD [53], which was applied to analysis of time-resolved FRET between DPH and NBD-phosphatidylcholine probes [54]. We summarize below more recent works on NBD probes, excluding NBD-labeled sterols (which are addressed in Section 7.2).

#### 3.1.1. NBD Fatty Amines (NBD-C_n_)

**Figure 3:**
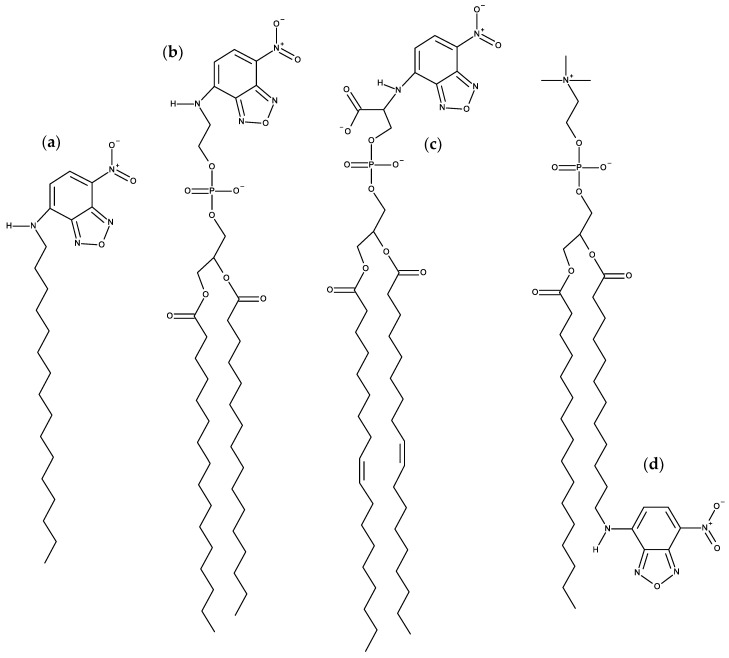
Structures of NBD probes. (**a**) NBD-C_16_; (**b**) *N*-NBD-DPPE; (**c**) *N*-NBD-DOPS; (**d**) C_12_NBD-PC.

Due to the small size of the NBD group and the sensitivity of its fluorescence on the polarity of the environment, this fluorescent moiety has been used to characterize the interaction of several amphiphiles with lipid bilayers. A recent example is the homologous series of NBD fatty amines (NBD-C_n_; Figure 3a) that was characterized both experimentally and by MD simulations [55,56,57,58,59], being an example of the complementarity and synergy between the two approaches. The ionization and photophysical properties of the homologous series NBD-C_8_ to NBD-C_16_ when associated with POPC bilayers was characterized experimentally and showed subtle variations along the series, suggesting a length dependent localization of the fluorescent NBD moiety [55]. MD simulation results for NBD-C_n_ (*n* = 4 to 16) in POPC bilayers supported the experimental observations and provided the molecular details behind the effects. The NBD fluorophore is located near the glycerol backbone/carbonyl region of POPC for all amphiphiles in the series, with the nitro group pointing towards the bilayer/water interface. Stable hydrogen bonds are established between the NBD alkyl amine and the POPC ester oxygens (mostly from *sn*-2), and between the NBD nitro oxygens and water. The small experimentally observed variations along the homologous series, (NBD-C_8_ presenting a larger fluorescence lifetime and smaller fluorescence anisotropy, while the opposite behavior is observed for NBD-C_16_ and intermediate alkyl chain lengths showing very similar results among them) where fully interpreted with the help of the molecular details provided by MD simulations. The position of the terminal methyl group of NBD-C_n_ (Cter) was found to be the most significant factor, with NBD-C_8_ being pulled down due to the location of its Cter near the POPC double bond, while NBD-C_16_ is pulled up to minimize interdigitation with the opposite POPC leaflet. A significant interdigitation is still observed for NBD-C_16_, which leads to a decrease in the amphiphile lateral diffusion coefficient. The effect of those amphiphiles on the properties of the POPC bilayer were very mild when compared to acyl-chain phospholipid analogues, indicating that those amphiphiles are good probes to characterize the properties of lipid membranes. The best match between the position of the amphiphile Cter and that of the POPC was observed for NBD-C_12_ and NBD-C_14_ [57].

The photophysical characterization of the NBD-C_n_ homologous series was also performed in Chol-enriched membranes, POPC:Chol 5:5 and PSM:Chol 6:4, both experimentally and by MD simulations [59]. It was observed that the NBD group is located closer to the membrane/water interface in those ordered membranes as compared with pure POPC bilayers, with its center of mass colocalized with or above the lipid glycerol group. This correlates very well with the decrease observed in the ionization constant of NBD alkyl amine (smaller stabilization of the neutral form) approaching the value observed for short alkyl chain NBD-C_n_ in the aqueous phase. In tandem with a shallower localization of the NBD group and the consequent higher exposure to water, the fluorescence quantum yield and lifetime is decreased, the latter partially explaining the higher fluorescence anisotropy observed. The large differences observed between the membranes with distinct lipid composition dominated the small variations observed among the amphiphiles in the homologous series, giving strong support to the use of those amphiphiles in the biophysical characterization of lipid membranes. Again, intermediate acyl chain lengths (*n* = 12 or 14) were identified as the most promising probes, due to the strong interactions established with the membrane and the absence of interdigitation with the opposite lipid leaflet.

**Figure 4:**
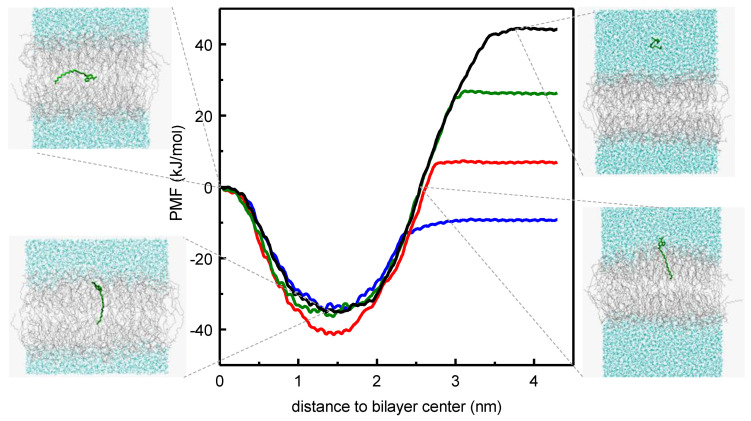
Comparison of PMF profiles calculated for different NBD-C_n_ probes (blue, red, green and black curves correspond to *n* = 4, 8, 12 and 16, respectively), pulling from the center of the bilayer to the water phase. Illustrative snapshots are shown for NBD-C_n_ at different locations along the reaction coordinate. Adapted with permission from [58]. Copyright 2014 American Chemical Society.

The kinetics and equilibrium of the interaction of the NBD-C_n_ homologous series with POPC membranes was also characterized experimentally and through MD simulations [55,56,58]. Umbrella sampling was used to obtain the PMF profile along the lipid bilayer, from which the equilibrium and kinetic parameters for interaction of the amphiphiles with the POPC bilayer may be obtained. The long alkyl chain was found to impose severe constraints on the MD simulation results for umbrella windows with the amphiphiles located near the water/membrane interface, requiring long simulation times to achieve convergence of the PMF. Notably, simulations performed with pulling of the amphiphile from water to the membrane did not achieve convergence even after simulation of NBD-C_16_ for over 100 ns in each umbrella window, while the pulling geometry from the bilayer center to the water achieved convergence after 25 ns. Another difficulty encountered was related with the use of the pull geometry distance, that led to significant deformation of the bilayer when NBD-C_n_ is located at intermediate positions in the membrane/water interface. This artifact was solved with the use of the pull geometry cylinder option, where the transversal location of the amphiphile is calculated relative to that of the lipids in a cylinder around the amphiphile. This systematic study is unique in the field and may provide guidance for further studies using long amphiphiles. From the converged PMFs (Figure 4), the difference in energy between the equilibrium location of the amphiphile in the membrane and that in the aqueous phase may be directly related with their relative water/membrane partition coefficients and the trend was in very good agreement with the results obtained experimentally. No significant energy barrier was encountered for insertion in the POPC membrane, in contrast with the results observed experimentally that show an insertion rate constant much smaller than that predicted for a diffusion-controlled process for *n* ≥ 12. This behavior may reflect limitations still observed in the sampling of the bilayer interface region, as previously reported for other long and very hydrophobic amphiphiles [60]. The energy barrier observed for the amphiphiles in the center of the bilayer is directly related with the rate of translocation between leaflets and the trend observed along the series was in good agreement with the results obtained experimentally, without significant effects of the length of the alkyl chain. An asset of this joint experimental and MD simulation study was the extension of the length of the alkyl chain in the homologous series to molecules not experimentally accessible, thus complementing and improving the predictive power of the work.

#### 3.1.2. NBD Phospholipid Probes

Following the work on acyl chain-labeled PC described at the top of this subsection, the next family of NBD phospholipids to be studied using MD were head-labeled phosphatidylethanolamines (*N*-NBD-PE). The dipalmitoyl derivative (*N*-NBD-DPPE; Figure 3b) was the object of a combined fluorescence/atomistic MD work by Kyrychenko et al. [61], aimed at calibrating these authors’ concerted fluorescence quenching and MD simulation depth distribution analysis procedure (see Section 3.4 for more details). The simulations presented in that article focused exclusively in the determination of the fluorophore transverse position in the POPC bilayer, which was centered at 1.44 nm from the center. This means that, despite its headgroup attachment, the fluorophore in NBD-diC_16_PE had a position similar to that of the previously addressed NBD-PC and NBD-C_n_, where it is attached to the apolar region of the molecule. A subsequent study by our group addressed the whole homologous series of fluorescent head-labeled phosphatidylethanolamines (*N*-NBD-diC_n_-PE) [62]. The longer-chained derivatives of *N*-NBD-diC_n_PE, with *n* = 14, 16, and 18, are commercially available, and widely used as fluorescent membrane probes. Properties such as location of atomic groups and acyl chain order parameters of both POPC and *N*-NBD-diC_n_PE, fluorophore orientation and hydrogen bonding, membrane electrostatic potential and lateral diffusion were calculated for all derivatives. Most of these probes induce local disordering of POPC acyl chains, which is on the whole counterbalanced by ordering resulting from binding of sodium ions to lipid carbonyl/glycerol oxygen atoms. An exception was found for *n* = 16 (*N*-NBD-DPPE), which displays optimal matching with POPC acyl chain length and induces a slight local ordering of phospholipid acyl chains. It was speculated that this packing and ordering abilities may be related to the reported favorable partition into liquid ordered phases of *N*-NBD-DPPE and the *n* = 18 derivative, *N*-NBD-DSPE (the latter should be better accommodated inside domains enriched in longer, predominantly saturated chains such as those of sphingomyelin inside lipid rafts). This MD study indicates a fluorophore location of around ~1.4 nm relative to the center of POPC membranes, therefore in close agreement with an earlier quenching determination [63] and the above mentioned simulation study [61]. However, like in other previously studied NBD lipid probes, the transverse distribution of the fluorophore of *N*-NBD-diC_n_PE is wide, spanning huge variations in water penetration, local dielectric constant, and hence, probe-perceived polarity. The environmental heterogeneity of this distribution was used to explain the complexity of the time-resolved fluorescence emission from membrane-inserted NBD probes. The attachment of the fluorophore to a PE head group does not alter its preferred location (near the glycerol backbone/carbonyl region), but changes its orientation (with the NO_2_ group facing the interior of the bilayer) and the pattern of H-bond interactions between the NBD amino group as donor and lipid acceptors, which in turn are probable causes for differences in the decay parameters of the two most common NBD phospholipid probes (*N*-NBD-PE and NBD-PC), and also affect the values of orientation factor averages relevant to FRET involving *N*-NBD-PE as a donor and/or an acceptor.

A multifaceted work on the behavior of NBD-labeled lipids, namely 1,2-dioleoyl-*sn*-glycero-3- phospho-L-serine-*N*-(NBD) (*N*-NBD-DOPS; Figure 3c), combined theoretical, experimental and simulation data to explain the true origin of the red-edge excitation shift (REES), i.e., the shift in the emission spectrum to longer wavelengths following excitation at the red edge of the absorption spectrum, that is displayed by NBD-lipid analogues in lipid membranes [64]. This study was driven by the fact that the understanding of important aspects of the photophysics of NBD remained incomplete, including the REES effect [65]. Density functional theory (DFT) and time-dependent (TD) DFT calculations of electronic structure provided good agreement with experimental absorption spectra and confirmed the experimental trends of increases in absorption and emission wavelengths for more polar solvents. The data clarified the traditional view that the photo-induced transition of NBD with lowest energy has more pronounced charge-transfer character compared with the second-lowest transition, and it was shown that both transitions are associated with electron displacements from the amino group in NBD to the heterocyclic ring and nitro group. More importantly, the value of the change of NBD dipole moment (Δμ) between the ground and first excited states was recalculated using up-to-date DFT methods, and verified that it is rather small (~2 D) [66,67,68]. This value was considered too small to be responsible for the REES of NBD-labeled lipids. Fluorescence measurements confirmed that the value of REES is ~16 nm for *N*-NBD-DOPS in DOPC vesicles. However, the observed shift was independent of both the temperature and the presence of Chol, being therefore insensitive to the mobility and hydration of the membrane. Furthermore, red-edge excitation leaded to an increased contribution of the fluorescence decay component with a shorter lifetime, whereas time-resolved emission spectra (TRES) of *N*-NBD-DOPS displayed an atypical blue shift following excitation. These observations were found not consistent with restrictions to solvent relaxation as the cause of the measured REES and TRES of NBD [65]. Instead, they pointed to the heterogeneous transverse location of probes as the origin of these effects. This was confirmed by the MD simulations. The calculated heterogeneity of the transverse location and hydration of NBD in the membrane correlated with the measured fluorescence lifetimes/REES [64]. Here, a deeper average location and larger extent of heterogeneity of transverse distribution (which was observed at lower pH) correlated with the experimentally measured longer average lifetime and increase in REES, respectively. The combination of theoretical and experiment-based techniques has led to a considerably improved understanding of the photophysics of NBD, in particular a reinterpretation of its REES.

**Figure 5:**
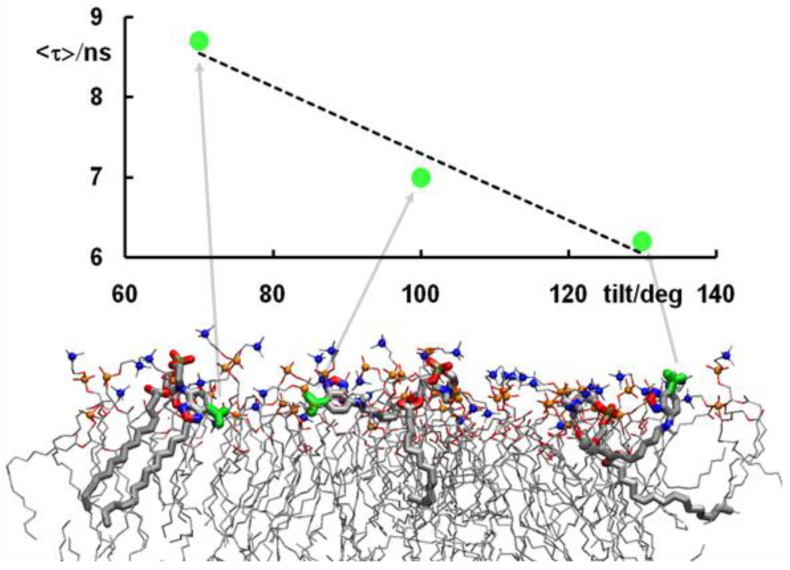
Intensity-averaged lifetimes <τ> obtained for different NBD probes in fluid PC bilayers, represented as a function of their most probable tilt of the NBD NO_2_ group relative to the bilayer plane, determined by MD simulation. Representative configurations (with the NO_2_ group highlighted in green) are shown to illustrate the preferred orientation of the different probes. *N*-NBD-DPPE, with its inward NO_2_ configuration (tilt angle <90°, left) has the longest average lifetime, whereas the opposite is observed for C_12_NBD-PC, in which the NO_2_ group is oriented towards the water medium (tilt angle >90°, right). An intermediate situation is observed for C_6_NBD-PC (center). Reprinted from [69] with permission from the PCCP Owner Societies.

More recently, it was shown how the orientation of the nitro group of NBD governs the fluorescence lifetime of nitrobenzoxadiazole (NBD)-labeled lipids in lipid bilayers [69]. This relation is represented in Figure 5. As mentioned above, irrespective of attachment to the lipid head group or hydrocarbon chains, the NBD fluorophore generally adopts a transverse bilayer location near the host lipid carbonyl/glycerol moieties. Still, considerable variability is observed in the measured fluorescence lifetimes, indicating that overall fluorophore location is not the determinant of NBD fluorescence properties. Combining fluorescence experiments and molecular dynamics simulations, it was shown that for two almost identical NBD probes, significant differences in fluorophore orientation and fluorescence lifetime are observed. Here, the direct comparison between the C_6_NBD-PC and C_12_NBD-PC (Figure 3d) probes, which reside in the same location of the PC bilayers, shows that different tilt angles of the NBD fluorophore correlate with higher exposure of the nitro group to the water phase (simulation) and higher degree of quenching (experimental). The orientation of the NBD fluorophore either exposes or protects the NO_2_ group from quenchers that can access the interfacial region where the NBD probe resides. Higher exposure of the nitro group results in higher quenching efficiency, be it by water molecules or other quenchers in the aqueous phase, and different fluorescent behavior of the fluorophore. The integration of these findings with other literature data, demonstrate a correlation between NBD orientation and fluorescence lifetime. The latter is longer when the NBD nitro group is predominantly oriented towards the bilayer interior, compared to probes for which it points to the water medium (Figure 5).

### 3.2. Prodan and Laurdan

**Figure 6:**
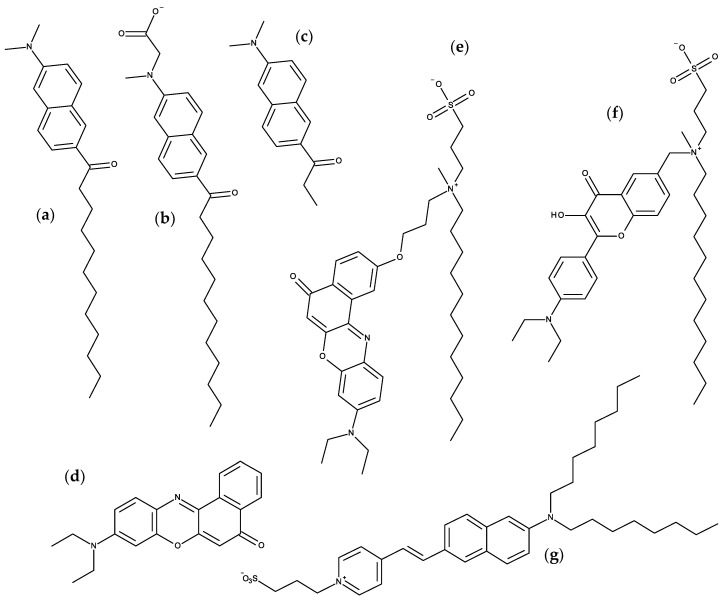
Structures of environment-sensitive probes. (**a**) Laurdan; (**b**) c-Laurdan; (**c**) Prodan; (**d**) Nile red; (**e**) NR12S; (**f**) F2N12S; (**g**) di-8-ANNEPS.

Prodan (6-propionyl-2-dimethylaminonaphthalene; Figure 6c) and its more lipophilic derivative laurdan (2-dimethylamino-6-lauroylnaphthalene; Figure 6a) are polarity-sensitive probes with unique features [70]. While laurdan distributes equally into ordered and disordered-like lipid phases (thus reporting on all lipid environments), it undergoes an emission spectral shift, from bluish in the ordered lipid phases to greenish in disordered ones. This is a consequence of the water dipolar relaxation following the large change in prodan and laurdan charge distribution upon excitation, with a marked increase in dipole moment. The differences in intensity in the blue and red sides of the emission spectrum of laurdan allow the definition of an useful observable to characterize membrane order, the so-called Generalized Polarization (GP) [71].

Barucha-Kraszewska et al. [72] carried out MD simulations of prodan and laurdan in water and in the presence of DOPC bilayers, both in the ground and excited electronic states. They found that laurdan penetrates more deeply in the bilayer than prodan, probably owing to the additional 9 carbons in its hydrocarbon chain, compared to the latter probe. Both molecules also displayed a more external transverse location in the excited state than in the ground state. The authors observed that, upon excitation, membrane-inserted molecules tend to move from ground to excited state transverse locations in the timescale of ~3–4 ns. On the other hand, the dynamics of solvent relaxation around the dye following excitation are in the order of ns for DOPC-inserted probe molecules, compared to ps in the water, in agreement with experimental observations. Additionally, dipoles of surrounding water molecules are more ordered around the instantaneous dye dipole in the excited state, compared to the ground state.

A follow-up study [73], combining MD simulations and quantum mechanical calculations, focused on prodan, took into account both planar and twisted excited state structures. Absorption and emission spectra were calculated for both forms using TD-DFT. Experimental emission energies agreed with the values calculated for the planar (cyclohexane) and the twisted (water and vesicles) structures of the excited S1 state of prodan in the gas phase. However, the oscillator strength of the twisted state is significantly lower than that of the planar configuration. The authors conclude that, in the bilayer, the fluorescence spectrum can be regarded as a combination of emission from both planar and twisted structures.

A subsequent work by the same research team compared laurdan with its carboxymethylated derivative, c-laurdan (Figure 6b) [74]. The additional polar group leads to a more stable and external ground state location of c-laurdan compared to that of the parent compound, and changes the fluorophore most probable orientation from close to parallel (for laurdan) to close to perpendicular (for c-laurdan) to the bilayer plane. Unlike laurdan, which changes average location upon excitation from <*z*> = 1.14 nm to <*z*> = 1.36 nm, c-laurdan does not change its fluorophore location upon excitation (<*z*> = 1.31 nm and <*z*> = 1.32 nm in the ground and first excited states, respectively). Dynamics of solvent relaxation around DOPC-inserted c-laurdan are similar to that observed for laurdan. C-laurdan was also studied by Osella et al. [75], who used QM/MM methods to calculate other optical properties, including one- and two-photon absorption spectra (similar for c-laurdan and laurdan, but exhibiting different environment sensitivity), as well as hyperpolarizability (double for laurdan compared to c-laurdan) and time-resolved fluorescence anisotropy in DOPC (pointing to more confined wobbling for c-laurdan, correlated with its more precise location).

A more recent effort utilized QM/MM to calculate absorption and emission spectra of laurdan in both gel phase DPPC and fluid phase DOPC bilayers [76]. Significant changes were observed in location (more external) and orientation (more aligned with the bilayer normal) of laurdan in the gel phase, compared to fluid membranes. Still, this resulted in only minor changes in the absorption spectrum, in qualitative agreement with experiments. Regarding the excited state, while the pattern of differences between gel and fluid is the same for location, the tilt distributions of the excited state fluorophore are more similar in the two phases, mostly parallel to the bilayer plane. This ultimately led to similar calculated emission spectra in the two phases, at odds with experimental evidence. Osella et al. [77] also addressed the behavior of laurdan in gel phase DPPC. They simulated the two conformers, corresponding to different orientations of the carbonyl oxygen respective to the naphthalene core. The two conformers adopt different orientations in the gel phase bilayer. No intermixing between the two conformations is possible, at variance with the behavior previously observed in fluid DOPC [75]. This study was continued in a more recent report, focused on optical properties calculated using QM/MM [78]. The solvatochromic nature of laurdan is apparent on the calculated optical properties, which can be affected by the presence of two different conformers. In the solid phase, the hindrance leads to more marked differences between conformers, in particular in the anisotropy decay (one of the conformers undergoes clear depolarization, while the other stays with anisotropy close to the fundamental limit value). At variance, because the interconversion between the two conformations is possible in DOPC and the environment is less viscous in the fluid phase, both conformers display clear emission depolarization in this environment.

Laurdan and prodan are used as probes of membrane order. But what if they perturb the bilayer, altering the local membrane order? In such a situation, the reported GP values could be affected by changes induced by the probe itself. To assess this possibility, Suhaj et al. [79] simulated prodan in liquid disordered DOPC and liquid ordered DPPC/Chol (7:3) at 300 K. Puzzlingly, from the computed order parameter profiles, these authors observed that prodan induced local disordering of disordered DOPC bilayers, while it manages to order an already highly ordered DPPC/Chol mixture, displaying a very external location (in the headgroup region) in this phase. Conversely, fluorescence microscopy experiments demonstrate that increasing the prodan:lipid ratio lowers the order of both phases. The authors ascribe this discrepancy to differing prodan localizations within the bilayer, and their effect on the hydration of adjacent lipids. It should be noted that the order parameter profiles shown in this work for DOPC molecules furthest from prodan, and therefore assumed to be free from perturbation, have a singular shape. The profiles are very different for the two acyl chains, missing the characteristic pronounced dip in carbons 9–10 (reported order parameter does not fall below ~0.15 for these atoms), and reaching unusually high values (>0.3) for the first 3–4 carbon atoms of the *sn*-1 chain.

### 3.3. Nile Red

The fluorescence probe 9-diethylamino-5*H*-benzo[α]-phenoxazine-5-one (Nile red or NR; Figure 6d), is a solvatochromic dye from the benzo[a]phenoxazine family. In solvatochromic dyes the position of the excitation and emission maxima are dependent on the polarity of their environment [80]. In case of Nile red, a shift toward shorter wavelengths is observed with the decrease of solvent polarity. Nile red has proven to be a useful tool for probing the lipid microenvironment. It was initially used for staining intracellular lipid droplets for detection by fluorescence microscopy or flow cytofluorometry [81]. Currently, it has many biological applications [82].

The position and orientation of Nile red in POPC bilayers was determined by calculating two dimensional Potential of Mean Force (2D-PMF) profiles as a function of distance and orientation with respect to the membrane normal [83]. The PMF profile indicates a strong preference for partitioning in the lipid bilayer as compared to the bulk water. Indeed, NR self-associates in water, a process that can be prevented by encapsulation in β-cyclodextrin [84]. The preferential position of the dye in all three systems considered, POPC and POPC containing oxidized lipids, is around 1.0 nm from the center of the bilayer. The dye is oriented in such a way that the largest principal axis is almost perpendicular to the bilayer normal. However, it can easily adopt other orientations. The orientation preference is not as strong as positional preference, especially for POPC, where the conformations with the diethyl amino group parallel to the bilayer normal are within thermal fluctuations of each other. Nile red is sensitive to the lipid environment and used for this purpose. Combined QM/MM models were used to calculate the emission spectrum of Nile red as a function of its local solvation environment. The results of QM and QM/MM computations are in qualitative agreement with the experimentally observed shift in fluorescence for the dye moving from aqueous solution to the more hydrophobic environment of the lipid interiors. A shift toward shorter wavelengths as the environment changes from pure water to a lipid/water bilayer was obtained. This was considered consistent with the experimentally determined blue shifts for Nile red upon transition from a polar (water) to a nonpolar (heptane) solution. However, eventually due to the complexity of the problem at hands, significant differences of absorption and emission values among different lipid environments were not identified.

The NR derivative NR12S (Figure 6e) was used to investigate the connection between the cytoplasmic machinery of apoptosis and the plasma membrane organization, by studying the coupling of caspase-3 activation and inhibition with phosphatidylserine exposure and the change of lipid order in plasma membrane. MD simulations of NR12S in disordered (DOPC:DOPE:Chol 3:1:2) and ordered (SM:Chol 2:1) were used to represent disordered and ordered phases of plasma membrane, respectively [85]. The analysis of the MD simulations concerned the location and orientation of the fluorophore, as well as its interaction with Chol molecules. The sulfonate group of the dye is anchored at the level of lipid head groups while its hydrocarbon tail is embedded into the membrane and behaves in the same way as the tails of the lipids. Despite possible limitations regarding the number of simulation replicates and simulation time for the systems addressed, distinct orientation patterns of the fluorophore were described in the lipid membranes. In the disordered phase both vertical and horizontal orientations were dominant and persist during different simulations. In the ordered phase, only the vertical orientation was considered dominant, although there is an evolution from horizontal to vertical during the single simulation carried out for this system. It was also found that the chromophore of the dye shows pronounced tendency to interact with Chol molecules, through transient contacts that form and disappear multiple times during the simulations. From the MD simulations it was suggested that the orientation of NR12S molecule is strongly conditioned by the lipid environment and interactions with Chol, which is likely to have strong influence on the fluorescence response. These results were used to complement experimental data and it was concluded that NR12S is a suitable tool to study early steps of apoptosis by means of sensing fine changes in the lipid order at the outer plasma membrane leaflet.

Nile red was used as a case study for rational design, through TD-DFT and MD simulations, of probe analogues for sensing in membranes [86]. All tested analogues showed a preferential location at ~1.0 nm from the center of the bilayer. All NR analogues showed some tendency to increase the order of the POPC acyl chains. However, this effect was more evident for the simulations with higher number of NR molecules. This calls the need for the use a low number of probe molecules in MD simulation, or comparable to those used in experiments, when studying their effects on the lipid membrane properties. 

### 3.4. Voltage and pH-Sensitive Probes

The membrane dipole potential, which stems from the dipolar components of the phospholipids and interface water, is a property of great importance in membrane biophysics. It accounts for the much larger permeability of a bare phospholipid membrane to anions than cations and affects the conformation and function of membrane proteins. However, its direct experimental measurement is difficult, and changes in fluorescence of voltage-sensitive dyes are often used to monitor variations in membrane dipole potential [87,88]. Curiously, the first MD article which targeted voltage-sensitive probes employed coarse-grained simulations to study the partition of dibutylaminostyryl-pyridinium butylsulfonate (Di-4-ASPBS) and its derivatives to POPC bilayers [60]. Since then, some other works have been published.

Warshaviak et al. performed MD simulations for two dipole potential fluorescent probes, F4N1 and di-8-ANNEPS (Figure 6g), in DOPC bilayers [89]. The probes are aligned with the membrane lipids with the charged group (quaternary amine in F4N1 and sulfonate in di-8-ANNEPS) located in the lipid polar head group region. The conjugated rings of di-8-ANNEPS span the DOPC monolayer, with the aliphatic tails located at the bilayer center. In contrast, the smaller F4N1 is entirely located within a single leaflet, including the short tails. The application of lateral tension to the membrane (up to 15 dyn/cm) increases the area per lipid, decreases the membrane thickness, and leads to a smaller dipole potential. Lateral tension leads to small variations in the location of the probes towards the bilayer center, with di-8-ANNEPS showing smaller effects due to anchoring of the aliphatic tails in the interleaflet region of the bilayer. The effect of lateral tension on membrane dipole potential obtained experimentally and from the MD simulations is in good qualitative agreement, although a much smaller variation is observed experimentally (5 versus 45 mV at 10 dyn/cm). The MD simulation results suggest that the discrepancy may in part be due to the small variations observed in the transverse location of the probes.

Di-8-ANNEPS was also the subject of a more recent work [90], in fluid DPPC, which verified that the probe aligns with the sulfonic group towards the membrane/water interface with the rigid aryl moiety tilted 19.3 ± 10.6° relative to the bilayer normal. In this study, some unusual choices of parameterization were taken, combining force fields AMBER GAFF for probe, CHARMM 36 for DPPC, and simple point charge (SPC) for water. Possibly related to these parameterization issues, a high water density (1.06 g/cm^3^) and excessive lipid order were observed (the latter may also reflect the choice of temperature just above the experimental main transition value). Non-linear optical properties were calculated, namely the hyper Rayleigh scattering response and the associated depolarization ratio (which are associated to the second harmonic light intensity and to the symmetry of the molecular scatterer, respectively), as well as the diagonal β*_zzz_* component of the first hyperpolarizability tensor, with *z* the direction normal to the bilayer. Notably, a large increase of the first hyperpolarizability of the dye was observed upon insertion into the bilayer.

Starting with the commercially available fluorescein-based voltage sensors (VF2.1.Cl, FluoVolt™), Kulkarni et al. carried out MD simulations to evaluate the location and orientation in the membrane, and gain insight towards the design of optimized new voltage sensors with improved sensibility [91]. It was found that VF2.1.Cl is poorly anchored in the membrane, presenting a broad distribution of orientations with an average tilt of 35 ± 17° relative to the bilayer normal. The orientation observed is a compromise between the favorable alignment of the aryl moiety with the lipid palisade, and the exposure of the single sulfonate group to the aqueous media. To improve the orientation of the probe along the membrane normal, the authors characterized by MD simulations a doubly sulfonated probe (dsVF2.1.Cl) and found that in fact, the tilt was decreased to 19 ± 10°. The improved properties of the double sulfonated sensors were confirmed experimentally, with 19% increase in the sensitivity to the transmembrane potential, as measured in patch-clamped HEK cells.

The use of optical probes for non-electrochemical pH determination is common in complex biological systems [92]. Diana et al. synthesized and characterized a new pH sensitive fluorescent molecule (5-(4-formyl-3-hydroxyphenoxy)-*N*,*N*,*N*-trimethylpentan-1-aminium bromide, PHABr), which changes from transparent to yellow at pH above 7 with increased fluorescence [93]. The experimental photophysical characterization was carried out in solution, and no relevant probe-membrane interaction measurements were performed. Still, MD simulations were performed to evaluate the location of the probe in membranes. It is found that the cation (pH < 7) is located just below the POPC head groups, while the zwitterion (pH > 7) is in the head group region, both parallel to the membrane surface.

### 3.5. Other Solvatochromic Probes

*N*-(5-Dimethylaminonaphthalene-1-sulfonyl) (dansyl) and the closely related *N*-(acetyl-aminoethyl)-5-naphthylamine-1-sulfonic acid (AEDANS) are versatile charge-transfer fluorophores for lipid and protein labeling. AEDANS, in particular, was extensively used by Hemminga and co-workers in a series of studies involving site-directed labelling of bacteriophage M13 major coat protein for the purpose of studying its lipid-protein interactions [94]. This group carried out an MD simulation study of various protein mutants along its sequence (containing a single transmembrane helix), in DOPC bilayers [95]. The conformations generated in the different simulation runs were then used for several analyses. These included orientations of the label relative to the transmembrane helix and explicit calculation of theoretical FRET efficiencies between the single protein tryptophan residue and the AEDANS label, without previous assumption of the orientation factor values, in general good agreement with experiments. Overall, the simulations suggest that the conformational space of the AEDANS label is only slightly affected by the presence of amino acid side-chains or lipids, advocating the use of AEDANS as a relatively inert environmental probe, structurally unhindered by the membrane lipids.

Coumarin-based probes have also been used to study membranes. A set of five such probes have been synthesized. These probes were reported to be “turn-off” sensors selective for Fe^2+^, 7-(diethylamino)-*N*-(1,3-dihydroxy-2-(hydroxymethyl)propan-2-yl)-2-oxo-2*H*-chromene-3-carbox-amide (AGD) [96]; and Cu^2+^, (*E*)-7-(diethylamino)-2-oxo-2*H*-chromene-3-carbaldehyde oxime (JB) [97]. The other three probes, 3-acetyl-7-[(6-bromohexyl)oxy]-2*H*-chromen-2-one (FM1), 7-[(6-bromohexyl)oxy]-4-methyl-2*H*-chromen-2-one (FM2) and ethyl 2-{7-[(6-bromohexyl)oxy]-2-oxo-2*H*-chromen-4-yl}acetate (FM3) were used as fluorescent probes for membrane and cell dynamics studies [98]. In all these works, MD simulations have been employed to characterize the behavior of the probes in POPC lipid membranes. MD simulations indicate that, in general, the coumarin moiety of these molecules interacts with the lipid bilayer at the level of the glycerol region. However, in all these studies, the order parameters of the POPC acyl chains are systematically too high. Therefore, the extraction of further details on the behavior of the molecules should be done with caution. In a previous study, Paloncýová et al. used coumarin as a test case for a methodological study about the convergence of free energy profiles of in a lipid bilayer [99]. Here, the most probable location of coumarin in DOPC membranes was determined at ~1.5 nm from the bilayer center.

Kyrychenko and co-authors studied the behavior of different excited state proton transfer probes interacting with lipid bilayers, combining steady state and time-resolved fluorescence studies with MD simulations. In a first report, indole-based probes 2-(2′-pyridyl)-5-methylindole (5M-PyIn-0) and 2-[2′-(4′,6′-dimethylpyrimidyl)]-indole (DMPmIn-0) were addressed [100]. Fluorescence emission from compounds is very sensitive to the protic nature of the solvent, being effectively quenched in water. From unrestrained simulations in POPC at 323 K, both compounds insert in the bilayer on a time scale of a few nanoseconds, to an equilibrium location of *z* = 1.2–1.3 nm from the center of the bilayer, in the upper acyl chain region, with indole rings aligned with the lipid tails. In the same time scale, the probability of hydrogen bonding between probe and water molecules decreases from essentially 100% before insertion, to below 10% at equilibrium. Free energy profiles across the bilayer were obtained with umbrella sampling, with global minima at −8.6 kcal/mol and −8.1 kcal/mol for 5M-PyIn-0 and DMPmIn-0, which compare with experimental values (obtained from partition curves) of −7.9 kcal/mol and −7.3 kcal/mol, respectively. A subsequent work concerned 2,6-bis(1*H*-benzimidazol-2-yl)pyridine (BBP), another polarity probe, which was studied using a very similar protocol [101]. In both studies, the large increase in emission intensity that accompanied probe partition into the bilayer correlated inversely with the probability of hydrogen bonding between probe and water. In turn, in the BBP study the authors verified that probabilities of hydrogen bonding calculated for varying probe transverse locations (corresponding to different umbrella sampling simulations) correlated with the calculated water density profile. A third study of these probes in bilayers addressed three different compounds with an identical 2-(2′-hydroxy-phenyl)-1,3-oxazole backbone, with either phenyl, biphenyl or phenanthryl moieties attached to (or fused with, in the case of phenanthryl) the oxazole ring, in fluid DPPC [102]. Fluorescence spectroscopy experiments revealed that all probes partitioned to lipid vesicles, exhibiting much increased emission intensity when inserted in the bilayer. In this case, for the most complex probes, preliminary calculations of potential of mean force profiles led to differences depending on the definition of the reaction coordinate. For this reason, probe location and distribution were ultimately determined solely from unrestrained simulations. As expected, increasing the size of the hydrophobic moiety (from phenyl to biphenyl to phenanthryl) leads to a more internal location of the fluorophore and indeed the whole probe. These compounds, with their rigid structure stemming from extended pi-conjugated systems, increased the order of the host lipid acyl chains, each of them most notably in the bilayer region where they preferentially locate. Another related probe, based on a 3-hydroxychromone skeleton, was simulated by these authors in another hydrophobic environment, dodecanethiol-coated gold nanoparticles [103], demonstrating favored location in the outer interfacial region of the nanoparticle.

**Figure 7:**
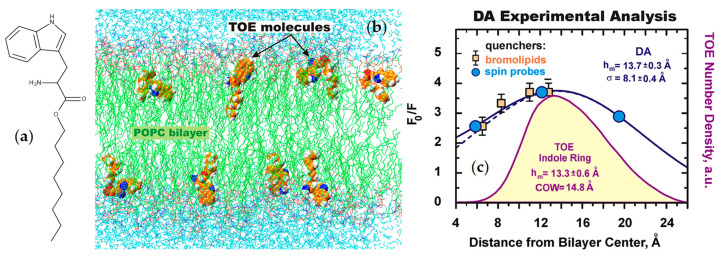
(**a**) Structure of tryptophan octyl ester (TOE). (**b**) Snapshot of an 8 TOE:288 POPC bilayer. (**c**) Comparison of experimental quenching ratios (data from bromolipid quenchers was scaled by a factor of 1.5 for visual comparison) and distribution analysis (DA) model parameters with TOE indole ring distribution obtained from MD simulation. *h*_m_: Gaussian fit center of quenching profile (experimental) or most probable location (MD distribution); σ: Gaussian fit standard deviation; COW: center-of-weight of the depth distribution. Adapted with permission from [105]. Copyright 2013 American Chemical Society.

The same group of authors subsequently developed an approach to determining the depth of fluorophores in the membrane combining fluorescence quenching and MD simulations. Actual quenching experiments for depth determination typically employ a set of brominated or aminoxyl spin label quenchers, with the quencher group attached at a distinct position in the molecule, leading to different quenching efficiencies, from which a fluorophore location (parallax method [63]) or distribution of locations (distribution analysis (DA) method [104]) is recovered. A first validation MD study used the model compound tryptophan octyl ester (TOE; Figure 7a,b) [105], for which the experimental DA treatment had been previously applied [106]. Depth-dependent collisional profiles were obtained by treating carbon atoms of acyl chain or headgroup of POPC as pseudoquenchers of TOE fluorescence, and (i) accounting for the overlaps of the *z* distributions of fluorophore and pseudoquencher, or (ii) calculating rates of collisions between TOE and these atoms from their close contacts during MD trajectories. The authors verified that the simulated quenching profiles were well fitted by a Gaussian probability density function of fluorophore positions, such as used in the DA method. DA of simulated quenching profiles based on the overlaps of *z* distributions estimated accurately not only the average penetration but also the width of the distribution. Experimental quenching profiles were much broader, possibly reflecting distributions of quencher depth and physical sizes of quenchers, as well as limitations in the number of quenchers used in each experimental study (Figure 7c). In the aforementioned study on *N*-NBD-DPPE [61], the analysis of experimental quenching data obtained using spin labeled lipids was refined using the quencher *z* distributions from separate MD simulations [107,108]. Profiles retrieved from both time-resolved and steady-state quenching measurements agreed very well with the fluorophore distribution obtained from MD simulation. Following this validation, the authors applied experimental DA to a preliminary study of the membrane penetration of two site selectively NBD-labeled mutants of diphtheria toxin translocation domain, which was expanded in a subsequent publication [109].

In addition to the location, the acknowledgment that the orientation of lipophilic probes can report on the physical and chemical properties of the biological membrane calls the attention to the importance of this particular characterization of behavior of the fluorescent probes, during the last years. The membrane orientational distribution of the 3-hydroxyflavone-based membrane dye F2N12S (Figure 6f) was investigated with a combination of polarization microscopy experiments, quantum chemistry and MD simulations [110]. The locations of several groups of the probe were obtained by calculation of the corresponding density profiles from the MD simulations. The ammonium nitrogen of the dye peaks at 1.30 nm from the bilayer center, below the average location of *sn*-1 and *sn*-2 carbonyl oxygen atoms of POPC; the density profile of the OH group of the dye acquires its maximum 1.25 nm from bilayer center, while the diethylamine nitrogen peaks much deeper at 0.65 nm; the SO_3_^−^ group of the dye has a density profile with a maximum at 1.60 nm, at the same distance from the bilayer center as the *sn*-2 carbonyl oxygen and below the peaks of the phosphate phosphorus and choline nitrogen. Regarding the orientation of the fluorophore, the experiments revealed a Gaussian-like distribution of the transition dipole moment of the dye, with a mean tilt angle of 53.2 ± 0.1° with respect to the bilayer normal and a standard deviation of 13.3 ± 0.6°. From the simulations, similar values of mean tilt angle of 48 ± 4° and a standard deviation of 13 ± 2° were obtained. MD simulations revealed that the aromatic rings adopted a tilted orientation with the diethylamine group directed toward the center of the bilayer, the SO_3_^−^ group embedded in the polar part of the membrane, and the twelve-carbon alkyl chain located among acyl chains of the POPC molecules. The good agreement between the experimentally and computationally obtained values cross-validates both approaches and gave confidence to the results obtained. This work was further extended to include the information from the DiI probe [111], and to explore the application of polarization microscopy to monitoring physiologically relevant processes in cellular membranes.

Another set of fluorescent probes that respond differently regarding their location dependent polarity and hydration at lipid/water interfaces is the homologous series of 4-aminophthalimide-based fluorescent molecules (4AP-Cn; *n* = 2–10, 12) having different lipophilicities [112,113]. MD simulations of this homologous series were carried out in the gel-phase of DPPC and fluid-phase of DOPC membranes [112]. The simulations were performed including only one probe molecule in the membrane, without replicates. Despite this statistical limitation, the MD results were discussed in comparison with experimental data from steady-state fluorescence and fluorescence quenching, allowing some plausible rationalization of the data. It was showed that besides positions, probe orientations also play an important role in defining the local dielectric environment by controlling the probes exposure to water at the interfaces, especially of the gel-phase DPPC bilayer. At the rigid DPPC/water interface, the position distributions of probes show a systematic shift from the shallow to deeper region of the bilayer, with the higher local polarity sensed by shallow probes compared to the probes located deeper inside. On the other hand, at the DOPC/water interface the position distributions of the probes are found to be much broader and show only subtle shifts among them. Similar observations were made for the orientation of the fluorophore, with larger variations in DPPC, while in DOPC the angle-distributions are significantly broadened showing only subtle shifts. It was concluded that the stepwise polarity-profile for the DPPC/water interface may suggest distinct regions near the interface, where solvation free energy controls the penetration and entrapment of solutes of different hydrophobicity in groups. It was argued that 4AP-fluorescence is highly responsive to a subtle change in the local environment at the interfaces formed by water and lipid bilayers of gel- and fluid-phases, which can eventually report on the location dependent polarity at the interfaces. The same authors reported a FRET study from the 4AP-Cn series as donor, which reside at different depths across the lipid/water interfaces, to the acceptor rhodamine-6G (Rh6G) molecules that stay in a water-rich region near the lipid headgroups [112,113]. In accordance with their previous description on the behavior of the 4AP-Cn series in lipid membranes [112], FRET from the series of 4AP-Cn donors to the Rh6G acceptor occurs in a peculiar stepwise fashion at the lipid/water interface of a gel-phase DPPC bilayer at room temperature. On the other hand, the same donor–acceptor pairs show only subtle but continuous donor depth-dependent FRET at the lipid/water interface of a fluid-phase DOPC bilayer.

4-(Diphenylamino)phthalonitrile (DPAP) is a recently synthesized probe that presents a tertiary amine electron donor and two nitrile groups acting as electron acceptor moieties, embedded in a π-extended conjugated system [114]. It displays solvatochromic (large red-shift and reduced quantum yield for high polarity solvents) and viscosity-dependent emission. A closely related probe, 4-(triphenylamino)-phthalonitrile (TPAP), was successfully tested in live cell imaging [115]. After careful parameterization of DPAP, it was simulated under the NVT ensemble in a variety of environments of different polarity and viscosity, including several solvents (acetonitrile, tetrahydrofuran, *o*-xylene and cyclohexane), a polymeric matrix and DOPC bilayers [116]. DPAP assumes an internal location within a bilayer leaflet (at an average distance of ~0.7 nm to the POPC phosphate atoms). As expected, its rotation within the membrane is much more hindered than in solution. The order parameter of the DOPC *sn*-1 acyl chain is noticeable reduced for the C atoms closest to the headgroup, while those of the atoms beyond the C9–C10 double bond are increased. Curiously, this effect is virtually inexistent in the *sn*-2 chain. A qualitative relationship between the measured fluorescence lifetime and calculated rotational correlation time was observed for the studied environments. The absorption spectra of DPAP in DOPC and other media were calculated, indicating moderate blue-shift in the bilayer compared to polar solvents.

Closely related to DPAP, the aggregation induced emission probes (*E*)-4-(2-(5-(4-(diphenylamino)phenyl)thiophen-2-yl)vinyl)-1-methyl-pyridin-1-ium (TTPy) [117] and (*E*)-4-(2-(5-(4-(diphenylamino)phenyl)-thiophen-2-yl)vinyl)-1-(3-(trimethylammonio)propyl) pyridine-1-ium (TTVP) [118] are also structurally based on a triphenylamine skeleton. These two dyes additionally include a thiophene group, an ethylenic bond and a charged pyridinium ring, and, in the case of TTVP, another positive charge, from a 3-(trimethylammonio)propyl group. The latter moiety constitutes the sole structural difference between the two probes. This rather subtle difference has a dramatic influence in the permeability properties of the probes, as TTPy can penetrate the plasma membrane and target mitochondria, whereas TTVP specifically stains the plasma membrane. A combined QM/MM approach was carried out to rationalize this crucial difference, as well as to predict other structures of related aggregation induced emission probes with behaviors similar to either TTPy or TTVP [119]. Free energy profiles of the two probes across a POPC bilayer were calculated, clearly displaying a shallower, closer to the interface well and a higher energy barrier for the more hydrophilic TTVP. Accordingly, permeability coefficients calculated with the inhomogeneous solubility-diffusion method [120] lead to a ~200 times higher value for TTPy compared to TTVP, in accordance with the experimental cell imaging observations. A mechanistic analysis revealed that the number of water molecules entering the membrane hydrophobic core is two times larger for TTVP than for of TTPy, resulting in the much higher energy barrier for translocation. Conformations extracted from the MD simulations were used for calculation of electronic structure and optical properties (emission spectra, excited state radiative decay rate constants). Finally, taking into consideration the findings of the computational study, four new aggregation induced emission probes—two analogues of TTPy and TTVP each—were synthesized, and their distributions were imaged in live cells, confirming the expected behaviors of mitochondria or plasma membrane stains, respectively.

## 4. BODIPY Probes

BODIPY (4,4-difluoro-4-bora-3a,4a-diaza-s-indacene) is a very popular fluorophore for probe development, owing to its relatively high photostability, neutral total charge, high molar absorptivity and fluorescence quantum yield, and sharp absorption and emission spectra that enable high compatibility in multicolor imaging [121]. The high hydrophobicity of BODIPY, which is often problematic in the fluorescence imaging of biological samples, may actually be advantageous in membrane studies, as it may provide the possibility of probing the inner regions of the bilayer, unlike other non-hydrocarbon probes. The first membrane BODIPY probe to be studied using MD was a Chol analogue [122], as described in Section 7.2 below (together with more recent studies on BODIPY sterol probes).

**Figure 8:**
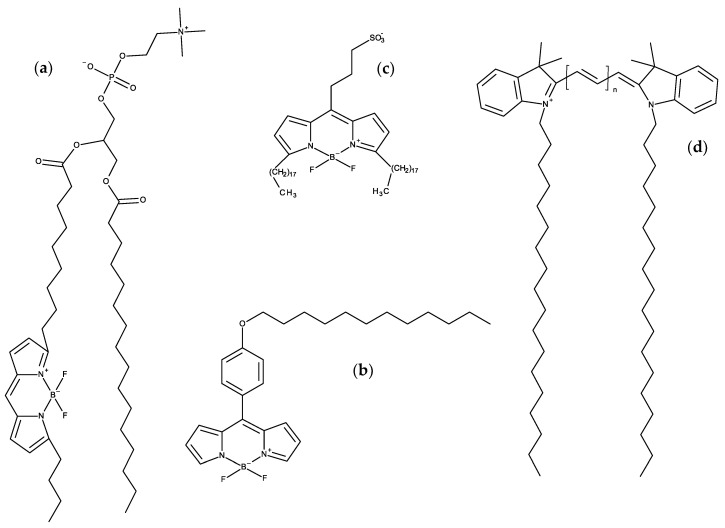
Structures of BODIPY and carbocyanine probes. (**a**) (1-hexadecanoyl-2-(4,4-difluoro-5-butyl-4-bora-3a,4a,diaza-s-indacene-3-nonanoyl)-*sn*-glycero-3-phosphocholine); (**b**) (4,4′-difluoro-4-bora-5-(p-oxododecyl)phenyl-3a,4a-diaza-s-indacene) (FMR-2); (**c**) 8-[(2-sulfonatoethyl)amino]-4,4-difluoro-3,5-dioctadecyl-4-bora-3a,4a-diaza-s-indacene (BNP); (**d**) generic structure of symmetric C18:0-chain carbocyanines. *n* = 1: DiIC_18_(3) or DiI; *n* = 2: DiIC_18_(5) or DiD; *n* = 3: DiIC_18_(7) or DiR.

A BODIPY-phosphatidylcholine with the fluorophore incorporated in the middle of the *sn*-2 chain (1-hexadecanoyl-2-(4,4-difluoro-5-butyl-4-bora-3a,4a,diaza-s-indacene-3-nonanoyl)-*sn*-glycero-3-phosphocholine; Figure 8a) was simulated in DPPC monolayers and bilayers at 323 K in the *NPAT* (constant pressure, area, and temperature) ensemble [123]. This study revealed that the BODIPY fluorophore has a very wide transverse location distribution, being able to exist in the geometric center of the bilayer, as well as near the headgroup region, with a maximum corresponding to the upper acyl chain regions for low lateral pressures (3 mN/m), or to the lower acyl chain for higher lateral pressures (40 mN/m). The same applies to the tilt, with low long axis tilts (corresponding with alignment with the bilayer normal) dominant for high lateral pressure, and wide distributions (including >90° angles, i.e., looped conformations) for low and medium (10 mN/m) values. This tendency of increased alignment with the bilayer normal for more ordered membrane systems correlates with the effect of increasing Chol content on bilayers containing BODIPY Chol analogue, described below [122].

Dent et al. [124] simulated three BODIPY probes, alkyl-tailed 4,4′-difluoro-4-bora-5-(*p*-oxodecyl)phenyl-3a,4a-diaza-s-indacene, a similar probe with two quaternary nitrogen atoms (thus introducing two positive charges), and a side-chain labeled Chol analogue (rotors 1, 2 and 3, respectively), in fluid DOPC and gel DPPC bilayers. The three probes showed similar transverse preferred locations in the upper acyl chain region for the fluorophore in DOPC, despite quite different locations of the other functional groups in each (the alkyl chain of rotor 1 inserting deeply in the bilayer, even penetrating the opposite leaflet; the charged chain of rotor 2 anchored at the headgroup region of the bilayer; and the Chol moiety of rotor 3 interdigitating to the opposite leaflet). Rotor 1 displayed two distinct conformations in gel phase DPPC, which were correlated to the observed bi-exponential fluorescence decay. Probe diffusion coefficients from simulation and experiment (using either fluorescence correlation spectroscopy or viscosities obtained from time resolved fluorescence data) were found with semi-quantitative agreement.

Steinmark et al. [125] used MD simulations to characterize another BODIPY-based molecular rotor, closely related with rotor 1 of the preceding paragraph, but with two additional methylene groups in the alkyl chain (4,4′-difluoro-4-bora-5-(*p*-oxododecyl)phenyl-3a,4a-diaza-s-indacene), or FMR-2; Figure 8b). Their simulations point to very small effects of FMR-2 on DOPC order parameters (slightly increased for first neighbor lipids), lateral diffusion coefficient (negligible changes) and hydration (~1 less water hydration molecule/first neighbor lipid headgroup). This probe was used to stain mitochondrial membranes and determine their viscosity (from fluorescence lifetime imaging microscopy (FLIM) data) in presence of different glucose concentrations. A subsequent work by the same group used FMR-2 to study liquid droplets, which were simulated and characterized by FLIM and time-resolved fluorescence anisotropy imaging [126]. Two dye populations were observed, one with shorter intensity lifetime and rotational correlation time, associated with lower viscosity, and another with longer intensity and anisotropy decay times, pointed to higher viscosity. The simulations also identified two populations with differing orientations in the membrane (~25° and ~55° average tilts of the boradiazaindacene-phenyl axis, relative to the lipid droplet monolayer normal). The authors identified each experimental lifetime component with one of the orientation modes observed in simulation, by noting that the component with highest amplitude (the shorter lifetime) should correspond to the most populated orientation (that with ~25° average tilt angle).

A combined MD/experimental study addressed the ability of BODIPY acyl chain-labeled monosialotetrahexosylganglioside (bdGM1) to emulate the behavior of GM1 in both ordered and disordered bilayers, including its binding to cholera toxin (CTxB), commonly used to label liquid ordered/raft domains [127]. Experimental binding studies revealed that although dissociation constant (*K*_d_) values are identical within the measured uncertainty for GM1 and bdGM1, in either DOPC (liquid disordered) or SSM/Chol (liquid ordered), the maximum number of binding sites available at the surface (*B*_max_) is reduced from near 100% for GM1 in both phases to ~64% and ~80% for bdGM1 in liquid ordered and disordered phases, respectively. This difference could not be explained from the MD simulations, as they revealed that headgroup orientation of GM1 is only very slightly altered from GM1 to bdGM1. Moreover, MD simulations in the presence of CTxB led to non-conclusive differences in hydrogen bonding interactions between the protein and the lipid headgroup for GM1 and bdGM1. Taking into account both experimental and computational observations, the authors suggest that the presence of the fluorophore on the acyl chain, rather than changing the headgroup geometry, largely excludes the bdGM1 molecules from the ordered membrane environments, and in turn usage of bdGM1 for studying GM1 behavior in cells can lead to invalid interpretation of experimental data.

**Figure 9:**
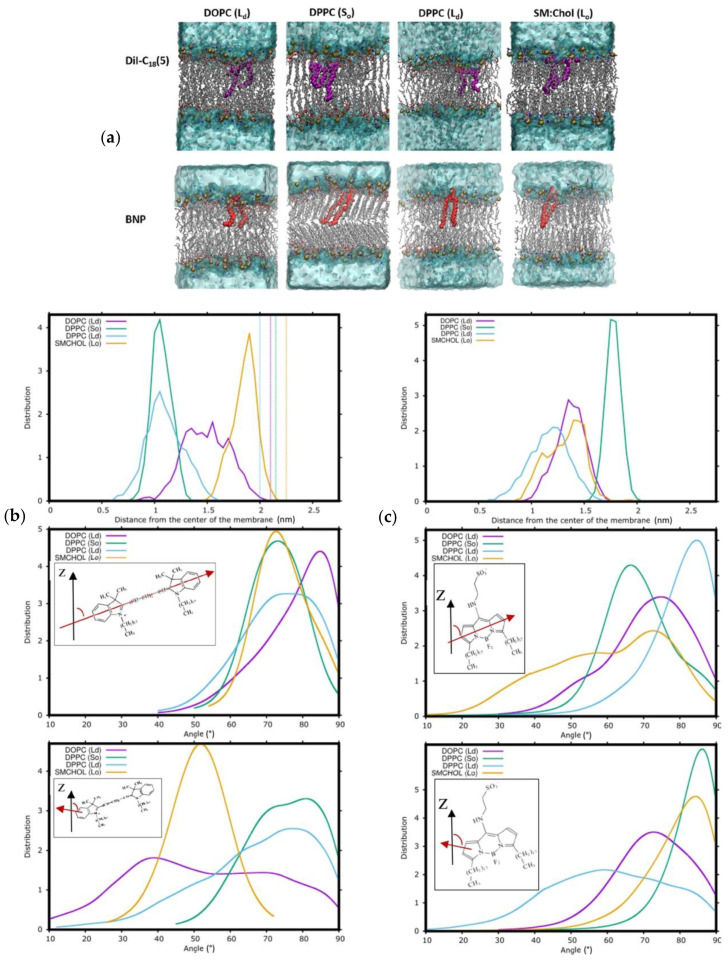
(**a**) Illustrations of the DiI-C18(5) (DiD) and BNP probes in different environments under investigation in the current study. (**b**) DiD in various membrane phases: (top) the position of the middle atom of the cyanine backbone of DiD along the *z*-axis expressed in terms of the distance from the center of the membrane, the dotted vertical lines denote the most abundant position of the phosphorus atoms; (center) the angle between the transition dipole moment and the *z*-axis; and (bottom) the angle between the axis perpendicular to the plane of the DiD molecule and the *z*-axis. (**c**) BNP in various membrane phases: (top) the position of the boron atom along the *z*-axis expressed in terms of the distance from the center of the membrane; (center) the angle between the transition dipole moment and the *z*-axis; and (bottom) the angle between the axis perpendicular to the plane and the *z*-axis. Reprinted with permission from [129]. Copyright 2018 American Chemical Society.

More recently, a *meso*-amino-substituted BODIPY probe, 8-[(2-sulfonatoethyl)amino]-4,4-difluoro-3,5-dioctadecyl-4-bora-3a,4a-diaza-s-indacene (BNP; Figure 8c) was prepared and characterized with different biophysical techniques, including MD simulations in DOPC bilayers [128]. Following this brief simulation study, this probe was addressed in more detail in several systems, including 2:1 PSM/Chol and solid ordered DPPC bilayers [129]. The main goal was to gain insights on the preference of BNP for more ordered lipid phases over liquid disordered ones. Gibbs free energy profiles were obtained by BNP using the *z*-constraint method, using the distance between the *z* coordinates of the B atom and the bilayer center of mass as reaction coordinate. These profiles displayed wells of −33 kcal/mol in fluid DOPC and liquid ordered SM/Chol, marginally deeper ones in DPPC gel (298 K) and significantly deeper in DPPC fluid disordered (323 K) phase. The corresponding *z* values were 1.2, 1.9, 1.1, and 1.5 nm for DOPC, DPPC gel, DPPC fluid, and SM/Chol, respectively. Energy barriers for translocation equaled ~10 kcal/mol for both DPPC systems and DOPC, and 14 kcal/mol for PSM/Chol. The BODIPY plane orients itself almost parallel to the membrane plane, especially for the ordered phases (Figure 9c). Anisotropy decays, calculated from rotational correlation functions, were highly hindered, with a long time limiting component ranging from ~40% for fluid DPPC to >90% for gel DPPC), consistent with experimental steady-state anisotropies of 0.15 and 0.35, respectively.

## 5. Carbocyanine Probes

Some of the most popular dyes used to study membrane structure are lipophilic dialkyl-carbocyanines, such as 1,1′-dioctadecyl-3,3,3′,3′-tetramethylindocarbocyanine (DiI-C_18_(3) or DiI) and 1,1′-dioctadecyl-3,3,3′,3′-tetramethylindodicarbocyanine perchlorate (DiI-C_18_(5) or DiD), among several others (see Figure 8d for general structure). These molecules have very high molar absorption coefficients and are used as membrane potential probes and as stains in cell studies. In our previous review [12], the atomistic behavior of DiI in DPPC bilayers was already described [130]. Since then, several works have been published, addressing mainly the local perturbation of these molecules in the lipid bilayers and their ability to distinguish between different lipid phases [129,131,132,133,134].

Ackerman et al. used MD simulations to investigate and better understand the perturbations in a fluid DPPC bilayer upon incorporation of three carbocyanine probes, DiI-C18:0, DiI-C18:2, or DiI-C12:0 [132]. The perturbations of DPPC by fluorescent probes within the same leaflet were nearly identical for all probes examined. The average locations of all probes were found in agreement with that obtained in previous DiI simulations in lipid membranes, with the fluorophore at roughly ~1.0–1.1 nm from the bilayer center. A 10−12% decrease in chain order was observed for DPPC in the solvation shell nearest the probe but smaller effects in subsequent shells, indicating that the probes significantly alter only their local environment, including the lipids directly across from the probe in the opposite leaflet. This opposite-leaflet effect was attributed to the protrusion of the probe hydrocarbon chains into the opposite leaflet. The local perturbation of lipids was extended to the orientation of their P-N vector caused by the chromophore location and charge. Overall, it was concluded that while DiI probes perturb their local environment, they do not strongly influence the average properties of lipid domains containing a few hundred lipids, and therefore can be used in this type of experimental studies.

The orientation of DiI in lipid membranes was also studied in detail employing a combined experimental and computational study of the orientational behavior of fluorescent dyes in phospholipid membranes [111]. In agreement with previous results [130,132], the chromophore of DiI was found to be located below the N and P atoms of the phosphatidylcholine headgroups. Both simulation and experimental results confirmed the predominantly parallel orientation of the DiI chromophore with respect to the surface of lipid membranes.

To disclose which of the coexisting membrane phases were being visualized by DiD and the above-mentioned blue BODIPY probe BNP [128], large-scale MD simulations and the *z*-constraint method to obtain free energy profiles were used [129]. Those calculations give an indication to explain why, at room temperature, both BNP and DiD probes prefer the gel (So) phase in DOPC/DPPC (2:3 molar ratio) and the liquid-ordered phase in DOPC/SM/Chol (1:2:1 molar ratio) mixtures. This study highlights significant differences between orientation and location and therefore in efficiency between DiD and blue BODIPY when they are used in fluorescence microscopy to screen various lipid bilayer membrane phases (Figure 9). Dependent on the lipid composition, the angle between the transition-state dipole moments of both probes and the normal to the membrane is found to deviate clearly from 90°, although this is the general preference. DiD was located in the headgroup region of the SM/Chol mixture, in close contact with water molecules, giving rise to a distinct behavior in simulations of fluorescence anisotropy in this membrane composition. This was not observed for BNP, which is located deeper in the membrane.

Following the previous work, Osella et al. [133] addressed how the combination of different (non)linear optical methods such as one-photon absorption (OPA), two-photon absorption (TPA), and second harmonic generation (SHG) as well as the study of the fluorescence decay time, leads to an enhanced screening of membrane phases using the DiD probe. Using hybrid QM/MM calculations and based upon the (non)linear absorption of the embedded probes, it was found that DiD can be used to identify between lipid bilayer phases. The TPA results finally confirm the particular location of the probe in between the polar headgroup region of the 2:1 SM:Chol mixture in the liquid ordered phase.

Recently, the behavior of several DiD probes in DOPC lipid bilayers were studied by MD simulations [134]. The different DiD probes were considered by varying the length of the attached alkyl tails and also the length of the cyanine backbone. During the simulations, all DiD probes entered the membrane and accommodated below the head groups region. Overall, the position and orientation of DiDs are heavily dependent on the length of their alkyl tails. For the long tail molecules, bearing 18 carbons per chain, the middle of the cyanine backbone located ~1.5 nm from the membrane center. Probes with longer cyanine bridge segment locate further deeper in the membrane. It was found that the orientation of the probes was dependent on the tail length. Considering the attachment of short methyl or propyl chains, DiD oriented with its tails facing the water, contrary to the ones with longer tails. This was reflected in the exposure of the nitrogen atoms towards the water environment. Using QM/MM calculations it was verified that the different local environment for differently oriented probes affected the one-photon absorption spectra, that was blue-shifted for the short-tailed DiD with respect to the DiD probes with longer tails. The studied probes were proposed to be useful for fluorescence microscopy and the described properties of added value for their experimental use.

Ardizzone et al. used DiI, DiD and DiR (1,1′-dioctadecyl-3,3,3′,3′-tetramethylindo-tricarbocyanine iodide) probes to obtain fluorescent organic nanoparticles (FONs) composed by quatsomes (QSs), formed by the self-assembly in water of cetyl trimethylammonium bromide (CTAB) and Chol in a 1:1 ratio [135]. The potential of the dye-loaded QSs as probes for biological imaging using stochastic optical reconstruction microscopy (STORM) was explored, including also the characterization of the DiI and DiD systems by MD simulations. Both DiD and DiI have their carbocyanine groups in contact with water, with the nitrogen atoms located in the hydrophilic region of the QS bilayer, in the region delimited by the head groups of the CTAB surfactants and the OH groups of Chol. The aliphatic chains were immersed inside the hydrophobic core of the QS. The orientation of the carbocyanine groups was on average parallel to the interface, despite some fluctuations with substantial deviations from the average angle. It should be noted that, the lamellar structure of the QS particles is different from that of the lipid membranes. In any case this is an interesting case of the use of MD simulations to characterize probe systems with potential biotechnological applicability.

## 6. Xanthene Probes

**Figure 10:**
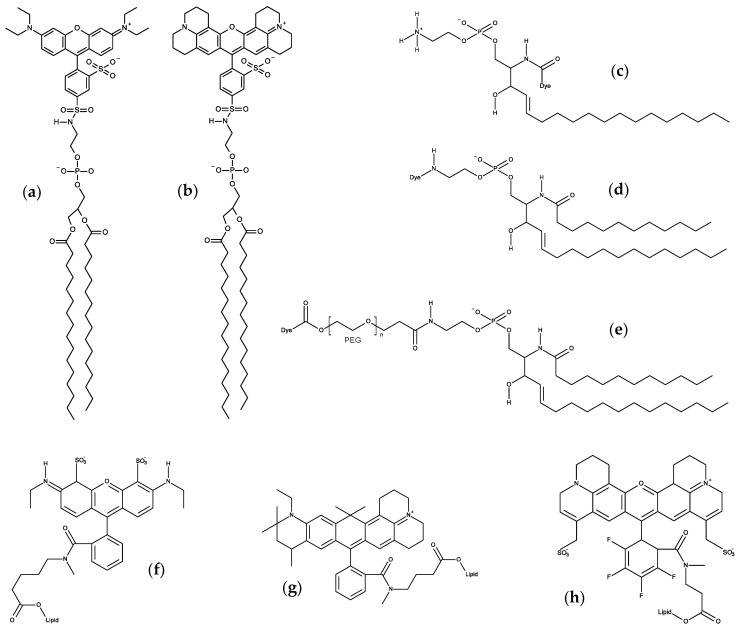
Structures of xanthene-based lipid probes. (**a**) Rhod-DPPE; (**b**) TR−DHPE; (**c**–**h**) general structures of acyl-chain-labeled (**c**), head-labeled (**d**) and PEG-linked sphingolipids (**e**), with fluorescent tags ATTO532 (**f**), ATTO647N (**g**), or KK114 (**h**).

Xanthene-based dyes, such as fluorescein and rhodamine derivatives, because of their convenient optical properties (brightness, long wavelength of excitation and emission, relative photostability) are popular fluorophores for numerous applications [136,137]. Being bulky polar groups, xanthenes are normally attached to the headgroup region of membrane lipids, to probe the lipid/water interface. Early MD simulation work on these compounds focused on Texas red (TR−DHPE [138]; Figure 10b) and lissamine rhodamine B sulfonyl (Rhod-DPPE; Figure 10a) [139], both in fluid DPPC. These were the first head-labeled fluorescent lipid derivatives to be simulated using MD.

Following on the simulations of 1 TR-DHPE:127 DPPC and 1 TR-DHPE:511 DPPC bilayers described in their earlier work [138], Skaug et al. simulated this probe in a more concentrated system, 24 TR-DHPE:488 DPPC [140]. This enabled a characterization of the previously suggested binding between the probe fluorophore and the phosphate group of the host lipid. The authors found that each TR-DHPE bound, on average, 1.2 DPPC molecules. This binding appears to be related to a dipole attractive interaction, that causes the DPPC headgroup to tilt more toward the bilayer normal, and slows down the rotation of DPPC molecular dipole (defined as the P-N axis). Another manifestation of this interaction is the slowed (by ~34%) lateral diffusion of TR-DHPE compared to DPPC. The authors invoked a theoretical model of the effect of cross-linking on lipid phase behavior [141] to predict that incorporation of ~1 mol% TR-DHPE could significantly expand the liquid ordered/liquid disordered phase coexistence region of DPPC/Chol mixtures, increasing the critical temperature of the phase diagram by ~5 °C.

More recently, Akhunzada et al. [142] studied a system similar to that of Kyrychenko [139], but employing doubly unsaturated probe and host lipid, Rhod-DOPE and DOPE, in the place of Rhod-DPPC and DPPC, respectively. Besides MD simulation, the authors carried out microspectroscopic measurements in giant unilamellar vesicles. Similarly to the earlier study, Akhunzada et al. observed increased area per lipid of DOPE in the presence of Rhod-DOPE, which agreed with laurdan GP and lateral diffusion measurements in doubly labeled (laurdan and photobleached Rhod-DOPE) DOPC vesicles, all pointing to a reduction in membrane order. Order parameters of the probe acyl chains were reduced compared to those of the host lipid. However, and similarly to what Skaug et al. observed for TR-DHPE, lateral diffusion of Rhod-DOPE was slower than that of host lipid, as observed both in MD simulations and fluorescence correlation spectroscopy data.

The authors ascribed this behavior to the larger hydrodynamic drag due to the bulky rhodamine B headgroup. The authors also investigated fluorophore aggregation, which is observed experimentally for large concentrations, by simulating a stable 8-Rhod-DOPE cluster in DOPC, with increased probe order within the aggregate and slightly lower order for the host lipids around it. Near the aggregate, the water density profile displayed a peculiar non-monotonic trend, including a significant increase (>25%) in water penetration inside the DOPC phosphate location. In turn, this led to a markedly altered electrostatic potential profile across the bilayer.

These authors published a continuation of this study, focusing on the temperature dependence (between 293 K and 320 K). They observed that the behavior of Rhod-DOPE paralleled that of DOPE in the same temperature range, i.e., increasing area/lipid values, while decreasing order parameter profile and bilayer thickness [143]. For all temperatures, the positions of the sulfonate and headgroup (NH-sulforhodamine B) probe groups roughly coincide with those of the phosphate and glycerol groups of DOPC, respectively. Regarding orientation, while the long xanthene axis has a unimodal distribution centered at ~90°, the perpendicular axis has a bimodal distribution, with predominance of inward fluorophore orientation, in agreement with the density profiles. These observations differ from those of Kyrychenko′s study of Rh-DPPC in fluid DPPC [139], which indicated a more outward orientation and external location of the fluorophore, and are actually more similar to those obtained for TR-DHPE by Skaug et al. [138].

These works revealed that headgroup-labeled probes exert local perturbation effects on bilayer properties, at similar distance ranges (~1 nm) to those previously reported for free probes such as DPH [29] or pyrene [39], or acyl-chain labeled lipids, such as NBD-PC [51]. A systematic effort to investigate the relative strengths and weaknesses of acyl chain labeling, headgroup labeling, and labeling based on poly-ethyl-glycol (PEG) linkers was carried out using sphingolipid derivatives of different xanthene-based dyes (the very hydrophobic ATTO647N, and the very polar ATTO532 and Abberior Star Red (KK114); see Figure 10c–h), in both liquid disordered (DOPC) and liquid ordered (PSM/Chol 2:1) bilayers [144]. The location of the different dyes depended on the dye, the point of linkage and the membrane phase. The more hydrophobic ATTO647N generally favored bilayer-inserted locations, while the more polar ATTO532 located in the headgroup/water regions, even when attached to the lipid tail. Generally, more external locations were observed upon dye attachment via a PEG linker and liquid ordered environments. Perturbations of host lipid properties (area per lipid, order parameter profiles) are more significant in the liquid ordered phase, and for acyl-chain linked dyes. Super-resolution imaging experiments in giant plasma membrane vesicles indicated that contrary to expectation, most probes preferred to locate in liquid disordered rather than in liquid ordered phases, with the notable exception of PEG-linked KK114 sphingolipid. KK114 probes in general also displayed lateral diffusion coefficients essentially identical to those of DOPC in MD simulations. However, and unlike most other studied probes, PEG-linked KK114 sphingomyelin displayed very similar diffusion coefficients measured for confocal (observation spot size *d* = 240 nm) and stimulated emission depletion recordings (*d* = 50–80 nm), indicating free Brownian diffusion and not showing the transient binding with slowly moving or even immobilized entities apparent for other probes. Therefore, and taking into account the small degree of perturbation induced by this probe, its use was recommended as a non-perturbing liquid ordered phase probe, with the note of caution that its usefulness to explore interaction dynamics with other molecules associated with biological membranes should be clarified in more detail.

Rhodamine fluorophores, such as tetramethyl rhodamine (TMR), are also commonly used to label peptides or proteins, including in the context of peptide- or protein-lipid interaction. This was the case for the sphingolipid binding domain (SBD) of the amyloid precursor protein, which was labeled with TMR [145]. The fluorescent peptide bound to isolated raft fractions from human neuroblastomas and insect neuronal cells, and thus could constitute a useful raft probe. More recently, a simulation study of this peptide was carried out, in interaction with POPC/Chol/SSM bilayers (15:10:4 mole ratio), containing varying amounts (0–12%) of ganglioside GT1b [146]. Both unlabeled and different versions of the labeled peptide, with different linkers (reflective of those used in previously published experimental data) were included in the sequence for MD. Insights on the peptide binding to GT1b were obtained from the simulations. The labeled peptide simulations were used in particular to establish whether the attached fluorophore affected the binding mechanism of SBD probes with gangliosides. Despite quantitative differences (the dye and linker moieties actually enhanced SBD peptide folding and stabilized binding with GT1b), this proved to not be the case.

## 7. Fluorescent Sterols

Chol is a major component of mammal plasma membranes. Although Chol itself is not fluorescent, a large number of fluorescent sterols are available, for use in spectroscopic and imaging studies [147,148]. Still, most of these probes have drawbacks regarding their optical properties and/or the extent to which they mimic Chol. Computer simulations, and MD in particular, are useful tools to clarify how these fluorescent sterols behave, compared to the native compounds.

**Figure 11:**
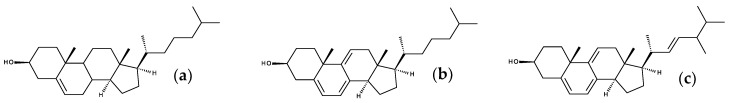
Structures of (**a**) Chol and intrinsically fluorescent triene sterols cholestatrienol (CTL, **b**) and dehydroegosterol (DHE, **c**).

The structure of Chol comprises three following fundamental moieties (Figure 11a): the rigid cyclopentaphenanthrene skeleton, the flexible isoprenyl side chain, and the polar hydroxyl group. Any of these parts of the Chol molecule may be modified for the purpose of obtaining fluorescent Chol analogues, and a number of probes are commercially available in each of these categories. However, to our best knowledge, no MD studies of steryl esters with fluorescent tags attached by esterification of the 3-OH group have been reported. Therefore, we will address below studies involving sterols with either an intrinsically fluorescent ring system, or a fluorophore attached to the alkyl side chain.

### 7.1. Intriniscally Fluorescent Sterols

#### 7.1.1. Cholestatrienol and Dehydroergosterol

Chol itself has a double bond between atoms 5 and 6 in the ring system. The existence of additional conjugated double bonds leads to a polyene-like fluorophore, which, depending on their number, may emit in the near-UV or even in the visible radiation range.

**Figure 12:**
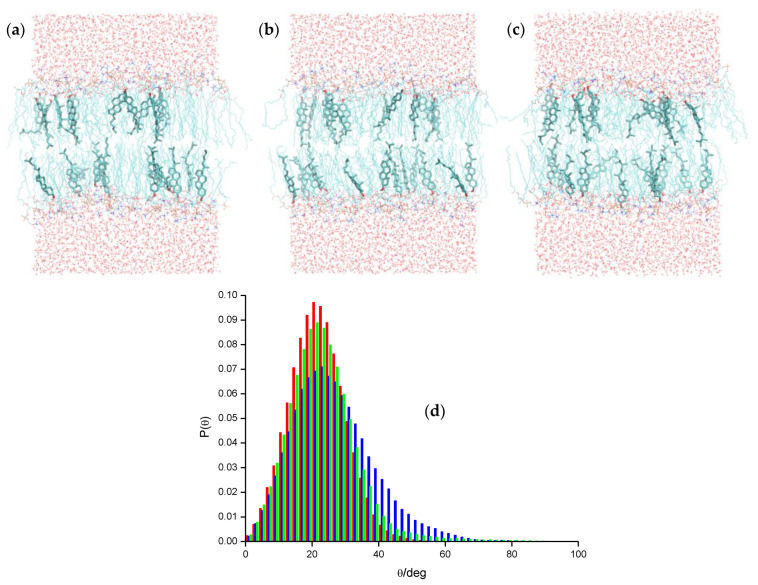
(**a**–**c**): Final structures obtained in the simulation of POPC bilayers with 20 mol% of (**a**) Chol, (**b**) CTL (**c**) DHE, respectively. Sterols are shown in thick lines, whereas water and POPC are displayed as thin lines. Cyan, blue and red colors are used for carbon, nitrogen and oxygen atoms, respectively (hydrogen atoms are not shown). (**d**) Probability density functions *P*(θ) of the angle between the sterol long axis and the bilayer normal for POPC/50 mol% Chol (red bars), CTL (green bars) and DHE (blue bars). Adapted with permission from [149]. Copyright 2013 American Chemical Society.

Cholestarienol (CTL; Figure 11b) is the fluorescent sterol which most resembles Chol, differing only in the 7–8 and 9–11 bonds, which are double, thus producing a conjugated triene chromophore, with absorption and emission maxima near 325 nm and 375 nm, respectively. Dehydroergosterol (DHE; Figure 11c) shares the same ring structure, but has a different side chain, identical to that of yeast sterol ergosterol. The behavior of these three sterols were compared in an MD study [149], using POPC as the host phospholipid. The compositions addressed in this study included essentially pure POPC with a minimal amount of inserted sterol (2 sterol:126 POPC molecular ratio), as well as 20 mol% and 50 mol% sterol. In the latter two compositions, different scenarios were considered: all sterol molecules were of the same type (either Chol, DHE, or CTL) or all of them were Chol except for two (one in each leaflet), which were replaced by either DHE or CTL. It was found that both DHE and CTL are adequate analogues, having similar transverse locations to that of Chol, orienting themselves upright in the bilayer (Figure 12a–c), and inducing ordering of POPC bilayers, albeit to extents slightly lower than that of Chol, in agreement with experimental measurements [150]. Probe rotational and translational dynamics were also similar to (generally slightly faster than) those of Chol. Of the two studied fluorescent sterols, CTL behaves closest to Chol (Figure 12d), also in agreement with experimental findings [150]. DHE, presumably due to its different side chain, shows slight but noticeable differences in sterol long axis tilts (larger for DHE; Figure 12d), area/POPC (higher for DHE), membrane thickness, POPC acyl chain order parameters (both lower for DHE), host lipid dynamics (faster for DHE), and distribution of Chol around each sterol (for 20 mol% Chol, radial distribution function values of Chol around DHE are generally lower than those around Chol and CTL).

A more recent study focused on DHE and its comparison with Chol, in 20 mol% sterol bilayers [151]. MD dynamics confirmed the finding of the previous study, adding some new insights on other properties, such as hydrogen bonding and charge-pair interactions (revealed to have identical patterns for DHE and Chol), and lateral pressure profiles (found to differ quantitatively but not qualitatively between Chol and DHE). Additionally, results from quantum chemical calculations and experimental fluorescence spectroscopy measurements were presented and discussed. Altogether, DHE was confirmed as having a similar behavior to Chol when inserted in membranes.

Both these studies confirmed CTL and DHE as suitable analogues of Chol in membranes. However, both of them also commented on the well-known limitations of these probes from a photophysical point of view, such as excitation and emission in the UV, low molar absorption, fluorescence quantum yield and photostability, which are serious deterrents to their use in imaging studies [148].

#### 7.1.2. Design Probes with Extended Double Bond Conjugation in the Ring System

**Figure 13:**
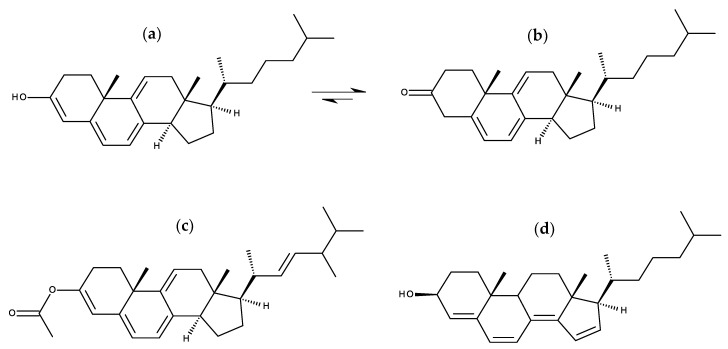
Structures of intrinsically fluorescent design tetraene sterols. (**a**) C4P (enol form); (**b**) C4P (keto form); (**c**) E4Pac; (**d**) unnamed promising tetraene sterol, which does not undergo enol-keto tautomerization [154].

Based on the structures of CTL and DHE, Kongsted and coworkers took the idea of extending the number of conjugated bonds in the ring system to develop fluorescent sterol probes combining their adequate mimicking of the behavior of Chol with suitable optical properties, namely enhanced brightness and emission in the visible wavelength range. In a first paper [152], these authors carried out quantum chemical calculations and MD simulations of three new Chol and three new ergosterol analogues, intended for use as fluorescent membrane probes. Besides the three double bonds of CTL and DHE (C5–C6, C7–C8 and C9–C11), the three tested compounds of each sterol type had a fourth conjugated double bond, each one at a different location (C12–C13, C1–C10 or C3–C4). Chol, ergosterol, CTL and DHE were also studied, for the sake of comparison. This was probably the first published paper where MD simulations of bilayer-inserted compounds were used to predict the behavior of novel membrane fluorescent probes yet to be synthesized. This study indicated that the compounds with C3–C4 fourth conjugated double bond (designated as C4P (Figure 13a) and E4P, for the Chol and ergosterol analogue, respectively) combined favorable electronic transition properties (predicted gas phase excitation and emission maxima at 364 nm and 461 nm, respectively, with high calculated oscillator strength values) with strong condensing abilities (comparable to those of the parent compounds). The quantum calculations of optical properties (both one- and two-photon absorption) were subsequently refined, using the sterol molecular configurations from the last frame of the DHE, CTL and C4P simulations, and the polarizable embedding model to account for membrane environmental effects [153]. Membrane effects on calculated one-photon absorption were modest for the three compounds, but more significant for two-photon absorption cross sections. The latter property scales with the square of change in permanent electric dipole moment Δμ, and was highest for C4P, which has a much larger Δμ value than DHE and CTL.

Following these computational studies, the C4P Chol analogue was ultimately synthesized and used for imaging in human fibroblasts [155]. However, upon synthesis, the fluorescent enol form undergoes tautomeric equilibration with the triene keto form (Figure 13b), and from electronic structural calculations of free energies of the two forms in vacuum, the authors estimated the keto:enol ratio to be ~6 × 10^3^ at 25 °C. For this reason, the authors also synthesized and studied the steryl acetate of E4P (E4Pac; Figure 13c), which kept the four conjugated double bonds. The experimental excitation spectrum of C4P in chloroform (λ_max_ = 325 nm) is similar to that measured for DHE (λ_max_ = 319 nm), confirming the predominance of the keto tautomer, whereas that of E4PAc has a large red shift (λ_max_ = 371 nm). On the other hand, emissions of both CP4 (λ_max_ = 423 nm) and E4PAc (λ_max_ = 436 nm) display large red-shifts compared to that of DHE (λ_max_ = 372 nm). Imaging studies revealed that both new fluorescent sterols can be taken up by the cells from albumin complexes, and E4PAc in particular was clearly distinguishable from cellular autofluorescence visible with a DAPI (blue) filter set. E4PAc was also found to bleach >2 times slower than triene sterol probes. Overall, this studied confirmed the utility of computational studies, including MD simulations, in the design of novel membrane probes.

The search for adequate ring-based polyene fluorescent sterols did not stop at this point, as the authors returned to that problem using a more systematic approach [154]. Following literature guidelines for functionality, a list of eight new polyene sterols was proposed. Of these eight compounds, three were excluded on the grounds of possibility of enol-keto tautomerization, with expected dominance of the less interesting keto form. Based on quantum chemical computation of optical properties in the gas phase, two tetraene molecules were selected as particularly promising, a first one with double bonds at C5–C6, C7–C8, C14–C15 and C16–C17 (larger Stokes shift, and emission at ~500 nm), and a second one with double bonds at C4–C5, C6–C7, C8–C14 and C5–16 (large oscillator strengths in both absorption and emission; Figure 13d). These two candidates were at last simulated in POPC bilayers with 30 mol% sterol. While the first compound led to noticeably lower acyl chain order parameter profiles, sterol long axis tilts and bilayer thickness compared to Chol (denoting reduced ordering capacity), the second one behaved almost identically to the parent molecule in all properties, and was thus proposed as a particularly good fluorescent sterol candidate.

**Figure 14:**
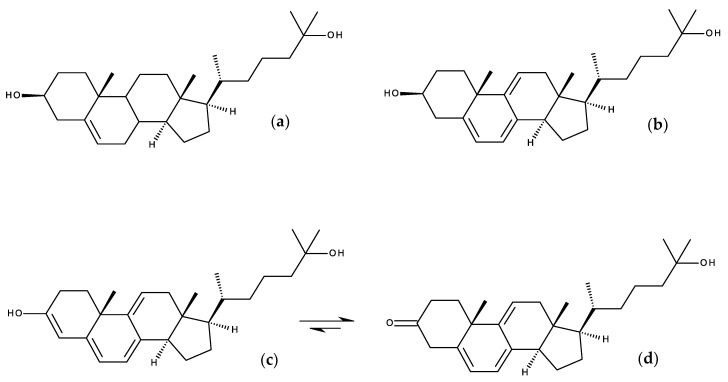
Structures of (**a**) 25-hydroxycholesterol and intrinsically fluorescent analogues: (**b**) 25-hydroxycholestatrienol (25-OH-CTL); (**c**) 25-OH-C4P (enol form); (**d**) 25-OH-C4P (keto form).

Previous to this latter study, the same research group had also used the same computational protocols to study 25-hydroxycholesterol (Figure 14a) and its previously synthesized fluorescent analogue 25-hydroxycholestatrienol (25-OH-CTL; Figure 14b), as well as the novel compound equivalent to C4P, with a 25-hydroxyl group (25-OH-C4P) [156]. 25-OH-CTL was confirmed as a good fluorescent analogue of 25-hydroxycholesterol, in terms of location, orientation, and effects on membrane ordering and thickness. The enol form of 25-OH-C4P (Figure 14c) also mimicked 25-hydroxycholesterol closely, which, taking also into account its predicted superior optical properties (red-shifted absorption, increased two-photon cross section) would suggest it as an adequate probe to mimic 25-hydroxycholesterol. However, similarly to the corresponding non-oxidized sterol, 25-OH-C4P is expected to exist mainly in the keto form (Figure 14d), which has optical properties characteristic of a triene, rather than a tetraene sterol, and also has propensity to adopt inverted orientation (with the carbonyl at C3 facing the interior of the bilayer, and the hydroxyl at C25 facing the interface). Thus, the authors ended up suggesting that it may be necessary to lock the probe in the enol form, possibly by acetylation such as described above for the non-oxidized sterols.

### 7.2. Side-Chain Labeled Sterols

**Figure 15:**
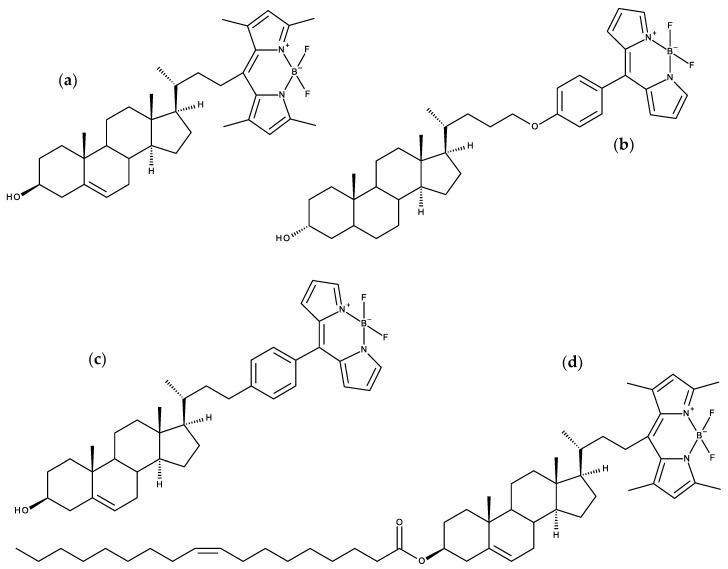
Structures of BODIPY side-chain labeled sterols. (**a**) TopFluor^®^ Chol; (**b**) phenyl-BODIPY litocholate-derived rotor with ether linkage; (**c**) phenyl-BODIPY Chol rotor with alkyl linkage; (**d**) TopFluor^®^ cholesteryl oleate ester (BODIPY-CE).

The works described in the preceding section illustrate the difficulty in designing intrinsically fluorescent sterols with adequate optical features. An alternative strategy consists in the attachment of a suitable fluorophore to the side chain. In this way, fluorescent sterols with good photophysical properties may be obtained; the main question is now how well they emulate the parent sterol molecules, and to this extent MD can provide considerable insights. This was illustrated in a combined live cell imaging and MD simulation study with the BODIPY Chol analogue 23-(dipyrrometheneboron difluoride)-24-norcholesterol (Figure 15a), also known as TopFluor^®^ Chol [122]. This probe was simulated in fluid bilayers of DPPC or PSM, as well as mixtures of the latter with Chol (4:1). It was found that TopFluor^®^ Chol was slightly less efficient than Chol in ordering lipid acyl chains, and that two main configurations of bilayer-inserted TopFluor^®^ Chol are possible: one similar to Chol, with upright orientation of the steroid ring system and deep fluorophore location; and another different from the parent compound, with tilted steroid ring system and shallow fluorophore position. While this latter configuration is significant at low total sterol content, it tends to vanish for 20 mol% sterol, indicating that TopFluor^®^ Chol is an especially good mimic of Chol for liquid ordered phases such as lipid rafts. This agreed with the imaging studies, which indicated that probe closely mimics the membrane partitioning and trafficking of Chol.

As described in Section 4, a BODIPY-based sterol rotor probe (Figure 15b), containing a phenyl group was characterized by MD simulation and fluorescence microscopy imaging ([124]; see Section 4). As mentioned above, the linker of this rather large construct (the BODIPY fluorophore group has a phenyl meso-substituent, which in turn is attached via an ether linkage to atom 24 of a litocholic acid residue) interdigitates across the DOPC bilayer, in order to place the polar BODIPY BN_2_F_2_ and sterol OH groups in the interface regions of the two opposite leaflets. In possible relation to this peculiar configuration, which is hard to accommodate in ordered bilayers, the imaging studies revealed that this sterol rotor probe displays a marked preference for the liquid disordered region of phase-separated DOPC/egg yolk sphingomyelin/Chol vesicles, at variance with the expected behavior.

A second BODIPY-Chol molecular rotor (Figure 15c), with an alkyl (rather than ether) linkage to C23 of a Chol residue (with unsaturated C5–C6 bond, unlike the first rotor mentioned in the above paragraph, which had an entirely saturated ring system), was synthesized and visualized in live cells, displaying significant co-localization with TopFluor^®^ Chol [157]. Very recently, this second rotor and TopFluor^®^ Chol were both parameterized according to a force-matching protocol, and simulated in both POPC and POPC/Chol 7:3 [158]. Both fluorescent sterols induced very little perturbation of POPC lateral molecular area and order parameter profiles, even for nearby POPC chains in the latter case. Sterol long axis tilts of the probes had similar distributions (somewhat wider for both compositions, slightly displaced—especially for TopFluor^®^ Chol-to higher angles in POPC) to that of Chol. On the other hand, the BODIPY fluorophore long axis (aligned with the transition dipole of the fluorophore) has very wide angular distributions in the phenyl-BODIPY rotor probe (even almost uniform in POPC), more so than TopFluor^®^ Chol, which has well defined peaks. Both results agreed with experiments using two-photon excited polarimetry in giant unilamellar vesicles [159]. Regarding transverse localization and distribution, the sterol moieties of both probes coincide with that of Chol in the POPC/Chol system, while the fluorophore has a wide *z* distribution for the phenyl-BODIPY rotor probe (probably due to its flexible linker) but a sharper location near *z* = 0 for TopFluor^®^ Chol. The rotation between the BODIPY and phenyl groups, crucial for the molecular rotor function, was observed in both POPC and POPC/Chol systems, slower in the latter by a factor of ~5–6. Overall, this study suggests that phenyl−BODIPY-Chol is a promising candidate for sensing Chol-induced membrane microviscosity in live cell studies.

Another study with BODIPY tags involved oleate steryl esters of both Chol (CE) and TopFluor^®^ Chol (BODIPY-CE; Figure 15d), and their interactions with a model of high density lipoprotein (HDL) [160]. This model had a hydrophobic core of 16 CE molecules (pristine HDL), of which 1 or 3 were replaced by BODIPY-CE in simulations of modified HDL. Compared to CE, BODIPY-CE had more external locations of the oleate tail and the ring system. This occurs because of the favored position of BODIPY between the acyl chains and the headgroups of the POPC molecules that surround the core. Free energy profiles, calculated for CE and BODIPY-CE using the replica exchange umbrella sampling method and defining the distance between the centers of mass of HDL and the Chol ring as reaction coordinate, led to essentially identical minimal values relative to the water phase, but larger differences relative to the HDL center and more external minimum locations for BODIPY-CE (reflecting its tendency to avoid very internal locations inside the HDL). On the other hand, lateral diffusion coefficients in HDL were ~4 times lower for BODIPY-CE, compared to CE. The authors concluded that while BODIPY-CE is a useful marker to mimic CE behavior in HDL, its replacement by a probe with no affinity for the hydrophilic surface region could lead to improvements in this regard.

**Figure 16:**
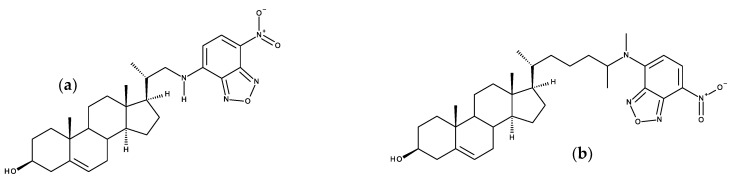
Structures of NBD side-chain labeled sterols. (**a**) 22-NBD- Chol; (**b**) 25-NBD-Chol.

A distinct study of side-chain labeled fluorescent sterols focused on two commercial NBD sterol probes, 22- and 25-NBD-Chol (Figure 16a,b, respectively) [161]. Although both probes have been employed to study the distribution and dynamics of Chol in different systems [147], important questions regarding their behavior remained open. From fluorescence quenching data, it was inferred that 25-NBD-Chol was deeply buried within the bilayer [63], while time-resolved fluorescence studies indicated that 22-NBD-Chol preferred Chol-poor disordered phases to Chol-enriched ordered ones [162], and NMR measurements suggested that both sterols could adopt upside-down orientations in bilayers [150]. MD simulations helped to clarify this matter, demonstrating that both 22- and 25-NBD-Chol are unable to mimic the most important features of Chol’s behavior in lipid bilayers [161]. Indeed, the fluorophores in both probes oriented toward the lipid/water interface, with a location similar to that observed in MD of other NBD probes (see Section 3.1). Consequently, the long sterol axis is no longer aligned with the membrane normal, and preferentially adopts orientations approximately parallel to the bilayer plane, especially for lower total sterol concentration. Interestingly, for very high sterol concentration (20 mol%), a significant proportion of fluorescent sterols adopts an upright conformation, possibly related to transbilayer sterol aggregation, reminiscent of that proposed in published experimental studies that showed spectral alterations (due to excitonic interaction) for large probe concentrations [163,164]. Because of this phenomenon, both fluorescent sterols (especially 25-NBD-Chol, possibly due to its longer chain) are capable of ordering the bilayer for the POPC/20 mol% sterol composition, but still considerably less so than Chol.

## 8. Conclusions

Looking back at a whole decade in MD simulation of fluorescent membrane probes, it could be said that this field has really come of age during the 2010s. The diversity of membrane probes that have been described is impressive. They range from small fluorophores to large molecular constructs, representative of all major classes of dyes. The fluorophores may be free, alkylated or attached to a natural lipid, either in the alkyl/acyl region or in the headgroup/polar region, or even attached to the side chains of membrane protein residues. Some of the studied probes have been developed recently, and a few of them had not actually been synthesized previously—quite importantly, MD simulation has also become a tool to test compounds prior to their actual synthesis. This makes perfect sense, as the power of parameterization tools, simulation codes and computer hardware has reduced dramatically the effort and time necessary for a preliminary computational screening of new compounds. Conversely, well established probes such as pyrene and DPH still attract interest, as novel aspects are considered.

In our previous reviews [12,13], we had identified several emerging trends. One of them—application of coarse-grained MD simulations, which was pioneered in the context of fluorescent probes late in the 2000s [60], seems to have stalled, and to our knowledge only NBD has been given the coarse-grained treatment more recently [69]. This is understandable as most of the information sought from simulation studies concerns finer grained properties, with atomic resolution as a requirement. If anything, whereas united-atom force-fields were previously the norm in MD simulation of membrane probes, use of all-atom force fields has been increasingly more common in recent times. Otherwise, we had anticipated that calculations of free energy profiles of the probe across the bilayer would become commonplace, and they are now routinely included whenever they may contribute to answering the questions at hand, as increased computational resources are now available to many researchers worldwide. However, it must be noted that calculations of free energy variations along the bilayer is not a trivial matter, and there are notable pitfalls that must be avoided, namely related to incomplete sampling of improbable states. This is noted in some of the works addressed above, which describe strategies to either establish adequate convergence of free energy profile [58] or use multiple reaction coordinates to tackle possible hidden energy barriers [83]. Ordered phases such as liquid-ordered or gel environments have considerably slower dynamics and require much longer simulation times than fluid disordered bilayers. For this reason, simulations in such systems were very expensive from the computational point of view, and thus relatively rare. Nowadays, this has become less of an issue, and, if required, these systems are now simulated without hesitation.

However, if there is a trend in recent simulation studies of fluorescent membrane probes, it is the combination of MD and quantum mechanical calculations to obtain a variety of properties of spectroscopic interest. The latter include absorption (including multiphotonic excitation) and emission intensity and spectral data, as well as fluorescence intensity and anisotropy characteristic times. These calculations are notably useful, as they allow rationalization of questions that had previously puzzled experimental researchers using older probes, as well as prediction of properties of novel ones. The application of these hybrid methodologies to the calculation of optical properties will undoubtedly constitute a mainstay of computational studies of fluorescent membrane probes in the following years.

Finally, it should be noted that, compared to previous years, a larger proportion of recent articles featuring membrane probe MD studies also include experimental work, either synthesis, spectroscopy, microscopy, or a combination of approaches. MD membrane probe simulations are therefore being widely accepted as an essential component in multi-technique approaches, complementary in nature, unique in detail, and well suited to reveal/rationalize subtle features that would otherwise go unnoticed/unexplained—the secret lives of these remarkable molecules.
